# Flubendazole exhibits anti-glioblastoma effect by inhibiting STAT3 and promoting cell cycle arrest

**DOI:** 10.1038/s41598-023-33047-9

**Published:** 2023-04-12

**Authors:** Barbora Vítovcová, Veronika Skarková, Radim Havelek, Jiří Soukup, Ananya Pande, Kateřina Caltová, Emil Rudolf

**Affiliations:** 1 Department of Medical Biology and Genetics, Faculty of Medicine in Hradec Králové, Charles University, Šimkova 870, 500 03 Hradec Králové, Czech Republic; 2 Department of Medical Biochemistry, Faculty of Medicine in Hradec Králové, Charles University, Šimkova 870, 500 03 Hradec Králové, Czech Republic; 3 The Fingerland Department of Pathology, Faculty of Medicine and University Hospital in Hradec Králové, Charles University, Sokolská 581, 500 05 Hradec Králové, Czech Republic

**Keywords:** Biochemistry, Cancer, Molecular biology, Molecular medicine

## Abstract

Glioblastoma multiforme (GBM) belongs to most aggressive and invasive primary brain tumor in adults whose prognosis and survival remains poor. Potential new treatment modalities include targeting the cytoskeleton. In our study, we demonstrated that repurposed drug flubendazole (FLU) significantly inhibits proliferation and survival of GBM cells. FLU exerted its effect by affecting microtubule structure and our results also suggest that FLU influences tubulins expression to a certain degree. Moreover, FLU effects decreased activation of STAT3 and also partially inhibited its expression, leading to upregulation of p53 signaling pathway and subsequent cell cycle arrest at G2/M phase as well as caspase-dependent cell death in GBM cells. These results suggest FLU as a promising agent to be used in GBM treatment and prompting further testing of its effects on GBM.

## Introduction

Glioblastoma multiforme (GBM) is the most common malignant primary brain tumor in adults, classified as grade IV glioma according to the World Health Organization (WHO)^1^. It is a highly aggressive and invasive type of malignancy^2^ characterized by a wide variety of genetic and epigenetic modifications leading to complex and heterogenous biological changes in GBM cells^1^. Current standard therapy protocols in clinical management of this tumor include primarily its maximal surgical resection (when possible), radiotherapy and/or chemotherapy with orally administered alkylating drug temozolomide (TMZ)^3,4^. Still, despite the use of these optimized treatment protocols, median survival of thus treated patients remains limited, being approximately 12–15 months after initial diagnosis^2,3^. Main reasons for therapy failure include the infiltrative growth and spread of GBM^2^, overall low TMZ brain concentration due to blood–brain barrier^5^ as well as hypoxic tumor microenvironment and related radio and chemoresistance of individual GBM cells^6^.

Limited current treatment options and poor prognosis in most cases of diagnosed GBM indicate the urgent need for new treatment modalities. These should reflect emerging or already studied GBM biomarkers proposed as targets for development of more effective drugs^7^. One group of these biomarkers concerns cellular cytoskeleton and its various components and basic constituents whose aberrant expression in GBM has already been acknowledged^8,9^. Besides select intermediate filament proteins such as vimentin^10^, it is mainly microtubules, in particular βIII-tubulin, which are dysregulated in GBM cells. βIII-tubulin is expressed in developing and mature neurons, but it is not present in adult glial cells. However, in glial tumors its expression associates with dedifferentiation, i.e. it increases with the tumor grade^8^. Moreover, βIII-tubulin appears to be more stable and localizes near necrotic areas too where its presence may contribute to cell survival^11^. In addition to βIII-tubulin overexpression, GBM cells are also positive for increased expression of γ-tubulin^12^.

Targeting microtubules belongs to one of the fundamental approaches in tumor treatment and due to the facts given above could be beneficial in GBM too^13^. The process of new anticancer drug development is costly and slow-paced, however, thus an alternative approach of drug repurposing seems to be potentially efficient and economical for finding new GBM treatment possibilities^14^. Flubendazole (FLU), benzimidazole veterinary anthelmintic, exerted anticancer effect in previous studies by modulating microtubule cytoskeleton in various cancer cell types, e.g., myeloma, breast cancer and neuroblastoma^15,16^. To this extent it might be a promising candidate for GBM drug repurposing. This presumption is further validated by the experimental evidence showing that FLU exhibited a significant antitumor effect on glioma cells associated with their G2/M cell cycle arrest^17^ and induced demise^18^.

Several mechanisms of FLU antitumor effect were proposed, including direct interference with microtubule dynamics resulting in cell collapse and death, activation of autophagy^19^ or the inhibition of signal transducer and activator of apoptosis 3 (STAT3) activation^20,21^. STAT3 is a transcription factor with very complex and multifaceted roles in several cellular and physiological processes including neural stem cell and astrocyte development^22^. Furthermore, it has been demonstrated that in glial malignant tumors STAT3 may play dual roles, being tumor suppressive or oncogenic, depending on the mutational profile of the tumor^23^. When significantly activated in GBM cells, it promotes cell proliferation and invasiveness^24^. In addition, constant activation of STAT3 negatively affects the expression of p53, subsequently downregulating p53-dependent signaling pathway^25^, allowing GBM cells to escape apoptotic process and leading to their survival.

This study was designed to investigate the effect of FLU on growth and proliferation of GBM stabilized cell lines and primary cells originating from patient’s tissue samples. Focus has been placed on the role of FLU-mediated STAT3 inhibition and resulting changes in p53 expression and associated cell cycle modifications.

## Methods

### Cell cultivation

A172, T98G, U118MG and U87MG cell lines were purchased from ATCC. A172 cells (ATCC® CRL-1620™) and U118MG cells (ATCC® HTB-15™) were managed in cultivation flasks in DMEM medium (LGC Standard); T98G cells (ATCC® CRL-1690™) and U87MG (ATCC® HTB-14™) were cultivated in EMEM medium (LGC Standard). Both media were supplemented with 10% fetal bovine serum (Gibco, Thermo Fisher Scientific) and 1% penicillin/streptomycin (Life Technologies, Thermo Fisher Scientific).

GBM clinical samples were obtained from patients undergoing surgery at University Hospital Hradec Králové. The study was approved by Ethics Committee, University Hospital Hradec Králové (Reference No. 201709 S13P) and patients gave their written informed consent. Cells were isolated by mechanic dissociation as described previously^26^ and all experiments were performed in accordance with relevant guidelines and regulations. GBM primary cultures (GBM49, GBM50, GBM57, GBM71, GBM72, GBM73) were grown in cultivation flask in cultivation medium: RPMI 1640 medium (Sigma-Aldrich) supplemented with 15% fetal bovine serum (Gibco, Thermo Fisher Scientific), insulin (100 IU/ml, Eli Lilly) and transferrin (2 mg/ml, Sigma-Aldrich). Cells were grown in humified atmosphere with 5% CO2 at 37 °C and subcultured every 3 days.

### Transfection of small interfering RNA (siRNA)

The siRNA against STAT3 (SignalSilence® STAT3 siRNA) and corresponding control siRNA (SignalSilence® Control siRNA—Fluorescein conjugate) were purchased from CellSignaling Technology. The transfection was performed on A172 and T98G cell lines using X-tremeGENE siRNA Transfection Reagent (Sigma Aldrich) according to manufacturer’s instructions. A172 and T98G cells were planted in 6-well plate (150,000 cells/well) and divided into three groups—control transfection, tested transfection and FLU (non-transfected) group. After 24 h cultivation the control transfection and tested transfection group were transfected using 50 nM control siRNA/siRNA against STAT3. The FLU (non-transfected) group cells were treated with 0.5 µM FLU. Cells were incubated for 48 h and then harvested for western blot analysis.

### Cytotoxicity assay

Viability of all stabilized cell lines and GBM primary cultures was evaluated using WST-1 assay. Cells were seeded in 96-well microtiter plate (7500 cells/well) and after 24 h cultivation were treated with TMZ (50 µM; 125 µM; 250 µM; 500 µM; 1000 µM) and FLU (0.5 µM; 1 µM; 2 µM; 3 µM; 5 µM) in DMEM or EMEM medium for up to 48 h. After the 48 h treatment, 100 µl of WST-1 solution (1:20 dilution) was added to the plate and the cells were further incubated for 2 h. At the end of incubation period, the absorbance was measured at 450 nm with 650 nm of reference wavelength using spectrophotometer Tecan Infinite M200 (Tecan, Switzerland).

### Cell proliferation assay by xCELLigence

xCELLigence Real-Time Cell Analyzer allows monitoring of the cell proliferation in real time using microelectrodes integrated in the bottom of E-plates. These microelectrodes measure electrical impedance and the relative change is displayed as representative unit called “cell index”. The measurement was carried in 16-well E-plate. First, 90 µl of medium was added to each well and the plate was inserted into the device for background measurement. Then, 100 µl of cell suspension (with 5000 cells) was added in quadruplicates in appropriate wells. Impedance was measured every 1 h for 24 h cultivation. After initial 24 h, cells were treated with 10 µl of treatment medium FLU in 0.5 µM and 2 µM concentration. Impedance was further measured every hour for 72 h.

### Phase contrast microscopy

The effect of flubendazole on the morphology and subcellular architecture of A172, T98G and GBM primary cultures was evaluated in standard tissue culture flask. Cells were exposed to 0.5 μM FLU and recorder under Olympus IX-70 phase contrast microscope using various magnifications after 24 h, 48 h and 72 h treatment.

### Immunofluorescence and image analysis

Cells were seated in cytospine chambers and were exposed to 0.5 μM FLU for 48 h. Afterwards cells were fixed with 4% paraformaldehyde (30 min, 25 °C), permeabilized with ice-cold methanol (10 min, − 20 °C) and blocked with 1% BSA in 1 × phosphate saline buffer (1xPBS) with 0.3% Triton X-100. Cells were rinsed twice with 1 × PBS between each step. After blocking cells were incubated with primary antibodies against α-tubulin 1:100 (Abcam), βIII-tubulin 1:50 (Cell Signaling) and β-actin 1:240 (Cell Signaling) at 4 °C overnight. After washing with 1 × PBS cells were incubated with Alexa Fluor goat anti-mouse 488 (1:250), Alexa Fluor donkey anti-rabbit 488 (1:250) and Alexa Fluor donkey anti-rabbit 546 (1:250) at 25 °C for additional 2 h and then labeled with DAPI (10 μg/ml). Cells were rinsed twice with 1 × PBS and mounted using ProLong Gold Antifade Mountant (Invitrogen-Molecular Probes) and examined under fluorescent microscope Nikon Eclipse E400.

### Western blot analysis

Confluent control and treated cells were washed with ice-cold PBS and harvested in ice-cold lysis buffer (50 mM Tris/HCl, 150 mM NaCl, 10% glycerol, 1% Triton X-100, 2 mM EDTA, 2 mM EGTA, 40 mM β-glycerolphosphate, 50 mM NaF, 10 mM sodium pyrophosphate, 200 µM Na_3_VO_4_, 2 mM DTT). The lysates were resuspended, and supernatants were obtained after 13,000 rpm centrifugation at 4 °C for 10 min. The quantity of total protein in supernatant was measured by BCA assay. Samples were boiled in SDS sample buffer at 95 °C for 5 min and 30 μg of total protein was loaded on SDS/polyacrylamide gel. After electrophoresis separation proteins were transferred to PVDF membrane (100 V, 90 min). Membranes were then blocked with 5% non-fat dry milk in TBST at room temperature for 2 h. Primary antibodies were diluted according to the manufacturer’s instruction in 2% non-fat dry milk in TBST or 2% BSA in TBST (monoclonal mouse anti-α-tubulin, 1:5000—Abcam; monoclonal rabbit anti-βIII-tubulin, 1:2000; monoclonal rabbit antit-STAT3, 1:2000; monoclonal rabbit anti-phospho-STAT3 (Tyr705), 1:2000; polyclonal rabbit anti-p53, 1:1000; monoclonal rabbit anti-cyclin B1, 1:1000; monoclonal mouse anti-GAPDH, 1:2000—Cell Signaling; polyclonal rabbit anti-p21, 1:2500; polyclonal anti-rabbit cdc2 p34, 1:2000—Santa Cruz Biotechnology) and membranes were incubated with primary antibodies at 4 °C overnight. After washing four times with TBST, membranes were incubated with secondary peroxidase conjugated antibodies (1:10,000, 2 h, 25 °C). Membranes were washed four times with TBST. The chemiluminescence detection was carried by ECL Prime Western Blotting Detection Reagent (Amersham, GE Healthcare Life Science) and relative quantification of chemiluminescence was evaluated with Azure c600 Imagining System (Azure Biosystems). All the original full-length blots or blots cut prior antibody hybridization are available in the Supplementary material S2 with membrane edges visible (if possible). The impossibility of providing full length blots or blots with visible membrane edges was caused by used antibody dilution or other limitations to the technology.

### Cell cycle distribution and internucleosomal DNA fragmentation analysis

Where the cell cycle distribution analysis is concerned, the cells were washed with ice-cold PBS and fixed with 70% (v/v) ethanol. In order to detect low- molecular-weight fragments of DNA, the cells were incubated for 5 min at room temperature in a buffer (192 mL of 0.2 M Na2HPO4 + 8 mL of 0.1 M citric acid, pH 7.8) and then labelled with propidium iodide in Vindelov’s solution for 1 h at 37 ºC. DNA content was determined using the flow cytometer CytoFLEX LX flow cytometer (Beckman Coulter) with an excitation wavelength of 488 nm. List mode data were analyzed using Kaluza Analysis 2.1 software (Beckman Coulter).

### Apoptosis luminescent assay

The A172 and T98G cells were incubated in 96-wells plate and treated with 0.5 μM FLU. After 4 h, 8 h, 16 h and 24 h incubation cells were harvested with caspase lysis buffer (50 mM HEPES, 5 mM CHAPS, 5 mM DTT). The activity of caspases 3/7, caspase 8 and caspase 9 were analyzed independently according to the manufacturer’s instruction using Promega Caspase-Glo assay (Promega).

### Immunohistochemical analysis of STAT3 and STAT3p705 expression

For the purposes of the study, all the GBM cases diagnosed between 2012 and 2015 at the Fingerland department of pathology were reviewed and the diagnosis was confirmed by single experienced neuropathologist. Only cases with representative amount of tissue without previous therapy were selected. Formalin-fixed paraffin embedded (FFPE) block of tumors were used for construction of 4 tissue microarrays (TMAs) employing TMA Master II system (3DHISTECH Ltd.). For each case, three representative tissue cores (1 mm in diameter) were transferred from donor blocks into the recipient block. In total, 4 TMAs were constructed and 3 µm tick tissue sections were cut for H&E staining and immunohistochemical staining. Heat induced epitope retrieval (HIER) was used for antigen retrieval. The detection of STAT3 (124H6, 1:400, Cell Signaling) was carried out o Benchmark Ultra stainer (Ventana/Roche), using Ventana OptiView DAB IHC detection kit. The detection of STAT3p705 (D3A7, 1:100, Cell Signaling) was carried out on Agilent/Dako Omnis (Agilent), using EnVision Flex detection kit. Both detection methods use avidin–biotin complex method with horseradish peroxidase as an enzyme and DAB (3,3'-diaminobenzidine) as chromogen. All slides were subsequently counterstained with heamatoxylin. The slides were first digitalized using Leica Aperio AT2 slide scanner (Leica Biosystems) and then evaluated with Aperio ImageScope software (Leica Biosystems). The evaluation of immunohistochemistry was performed by a single neuropathologist; percentage of positive cells (cytoplasmic and nuclear positivity of STAT3 and nuclear positivity of STAT3p705) and staining intensity (1—weak, 2—moderate, 3—strong) were recorded. Only tumors represented by the area of at least one core were included.

### Statistical analysis

Statistical analysis was carried out using GraphPad Prism 6.0 Software. Two-way ANOVA was used and was followed with Sidak’s multiple comparison test. Statistical significance corresponds to the level of reliability up to *p* < 0.05. The half-maximal inhibitory concentration IC50 was calculated using nonlinear regression by GraphPad Prism 6.0.

## Results

### FLU inhibits GBM cells proliferation

**Figure 1:**
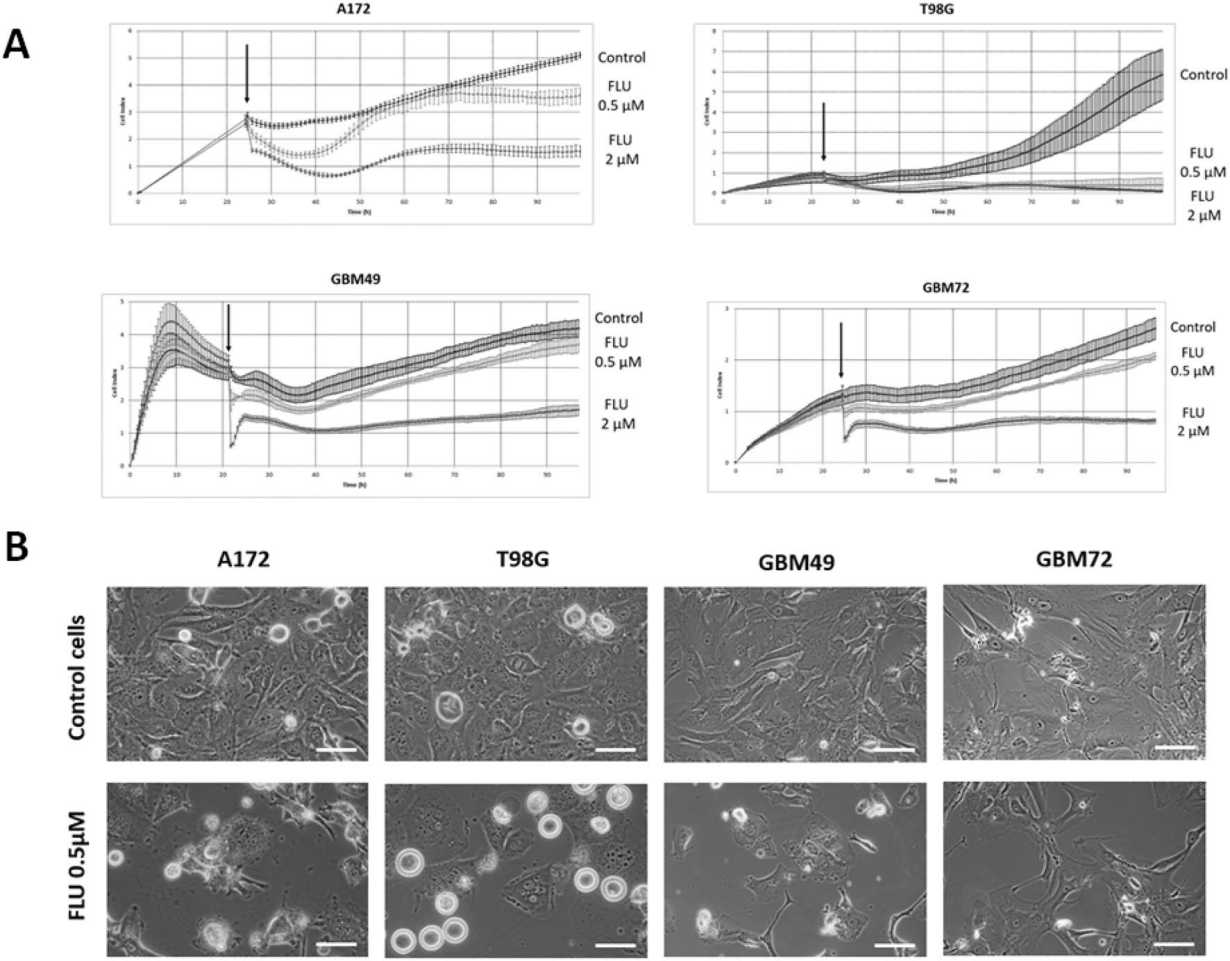
The effect of FLU on proliferation of GBM cells. (**A**) Proliferation of A172, T98G, GBM49 and GBM72 treated with 0.5 µM and 2 µM FLU during 72 h monitored via xCELLigence system. Each value is expressed as mean ± SD from four replicate wells. Figure shows representative curves from two independent measurements, arrow indicate the point of treatment. (**B**) Morphological changes and multinucleation caused by FLU 0.5 µM in GBM cells. Cells were treated accordingly and observed during up to 72 h using phase contrast microscopy, shown images represent 72 h time point. Magnification 400 ×, scale bar 20 µm.

The effect of various FLU concentrations on viability and proliferation on GBM cells was evaluated using WST-1 assay during 48 h exposure. Four GBM cell lines (A172, T98G, U118MG and U87MG) and nine GBM primary cell cultures (GBM45, GBM49, GBM50, GBM51, GBM59, GBM64, GBM67, GBM72 and GBM73) were used for initial evaluations. All tested cell lines were also treated with various TMZ concentrations used as reference. FLU-mediated inhibition of cell viability and proliferation proved to be more effective than in case of TMZ in all stabilized cell lines as well as in GBM primary cultures. The half maximal inhibitory concentration (IC50) of FLU varied between 0.6 and 3.9 µM, while the lowest IC50 for TMZ was 1000 µM (Table 1). Based on varying sensitivities of employed cells to TMZ and FLU, two cell lines (A172 and T98G) and two primary cultures (GBM49 and GBM72) were selected for further experiments with xCELLigence system. The results obtained from this continuous, real-time cell analysis confirmed the significant inhibitory effect of FLU on GBM cells proliferation at even lower drug concentrations (Fig. 1A). Based on these results, 0.5 µM FLU concentration was selected to be used in most subsequent experiments.

**Table 1:** The half minimal inhibitory concentration (IC50) of TMZ and FLU treatment in GBM stabilized cell lines (A172, T98G, U118MG, U87MG) and GBM primary cultures (GBM49, GBM50, GBM57, GBM71, GBM72, GBM73) treated for 48 h.

	IC50 [µM]	
	TMZ	FLU
A172	1840.0	3.959
T98G	3255.0	2.181
U118MG	3056.0	1.329
U87MG	2699.0	1.712
GBM49	1601.0	0.661
GBM50	990.3	2.762
GBM57	5480.0	2.937
GBM71	25,852.0	2.084
GBM72	11,236.0	3.376
GBM73	1300.0	1.753

Cell proliferation was measured using WST-1 assay and IC50 values were calculated using GraphPad 6.0.

### FLU has a significant effect on cell morphology

Two GBM cell lines, A172 and T98G, and two primary cultures, GBM49 and GBM72, were treated with 0.5 µM FLU and their morphology as well as behavior were observed using phase contrast microscopy during up to 72 h of exposure. In comparison with untreated controls, FLU exposed cultures were primarily inhibited in their growth and reproduction which became apparent already after 24 h of exposure (attached as supplementary material S1). Simultaneously, cells started to change patterns of their organization, their size and shape, generally tending to form large multinucleated complexes. These complexes as well as individual cells regardless of their size detached from substratum, rounded up and formed various blebs or bleb-like protrusions. The timing and extent of the described changes although most extensive at 72 h of treatment were nevertheless asynchronous, heterogeneous and cell line/culture specific (Fig. 1B).

### FLU causes marked changes in microtubule cytoskeleton

**Figure 2:**
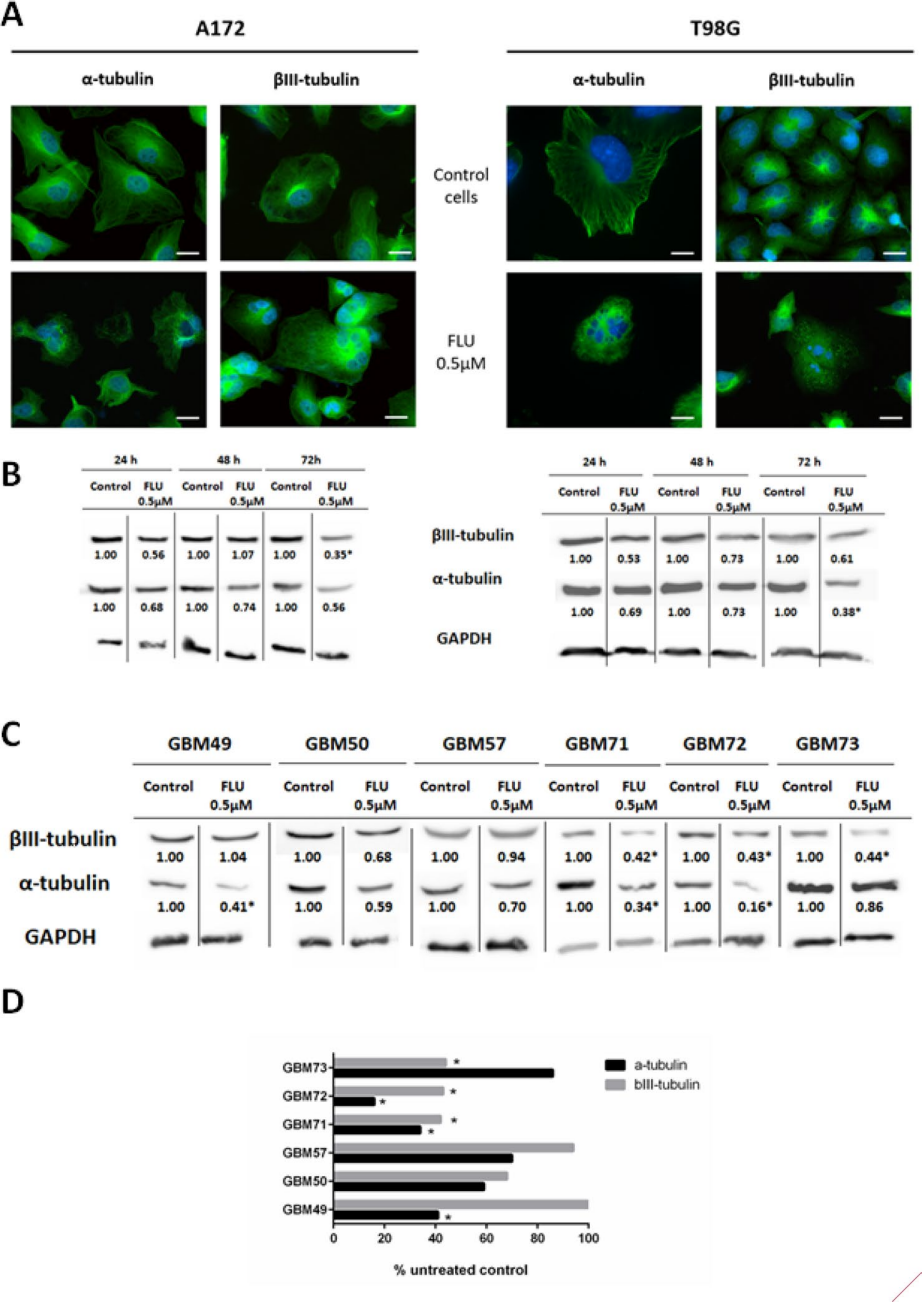
The effect of FLU on α- and βIII-tubulin in GBM cells treated with 0.5 µM FLU for 48 h. (**A**) The changes in α- and βIII-tubulin in A172 and T98G observed via fluorescence microscopy (magnification 600x, scale bar 10 µm) and (**B**) measured with western blotting. (**C**) The changes in α- and βIII-tubulin in GBM primary cultures measured by western blotting. All the western blotting results were processed and original blots are presented in supplementary files S2. (**D**) Quantification of the changes in α- and βIII-tubulin expression on protein level obtained via western blotting and expressed as percentage of untreated control. *p ˂ 0.05 vs untreated control.

As microtubules are the main cellular target of FLU, our next experiments investigated the effect of the drug on expression, localization, and structure of tubulins subunits in GBM cell lines A172 and T98G. Compared to control specimens, FLU treatment induced microtubule disorganization, with both α and βIII tubulin filaments being disrupted and twisted. Also, an overall pattern of microtubule network organization changed in a cell type specific manner as visible in multinucleated cells in particular. (Fig. 2A). Analyses of tubulin expression via Western blotting in exposed cells demonstrated that in A172 cell line α-tubulin and βIII-tubulin levels decreased after FLU treatment at three tested time intervals (24, 48 and 72 h of FLU exposure) (Fig. 2B). In view of these results, we also examined the changes in protein expression of α- and βIII-tubulin after FLU treatment in GBM primary cultures and as well as in previously tested cell lines. Data shown in Fig. 2C,D indicate that the expression of individual or both tubulin types (i.e. α ad βIII) was decreased after 48 h of FLU treatment.

### FLU lowered the STAT3 activation and arrested the cells in G2/M phase

**Figure 3:**
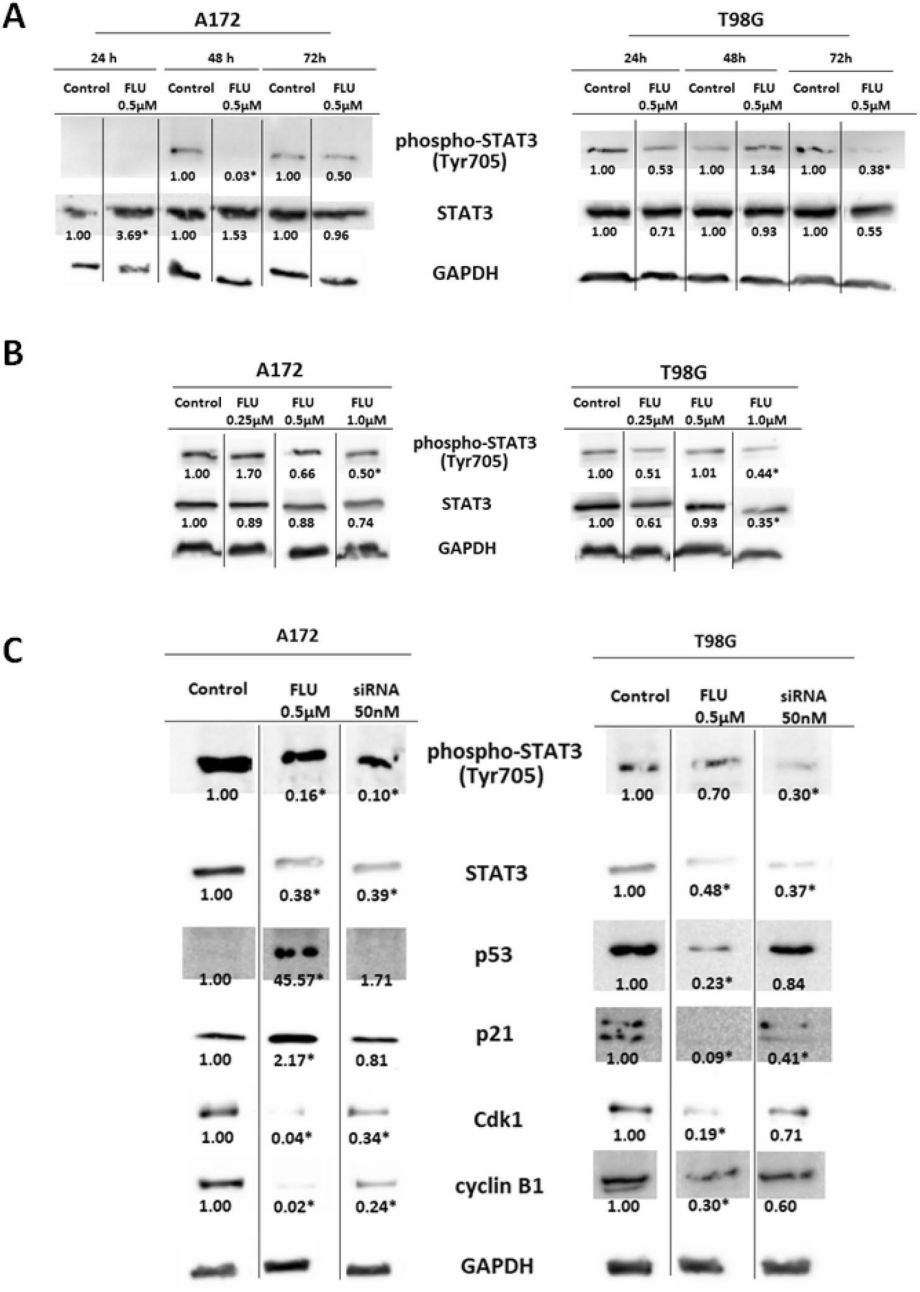
The effect of FLU on STAT3 expression and activation in A172 and T98G cells. (**A**) The time dependence of FLU effect on STAT3 and phospho-STAT3 (Tyr705) expression. Cell were treated with 0.5 µM FLU and harvested in three time points—24 h, 48 h and 72 h after treatment. (**B**) The concentration dependence of FLU effect on STAT3 and phosphor-STAT3 (Tyr705) expression. Cell were treated with three different FLU concentrations (0.25 µM, 0.5 µM and 1 µM) and harvested after 48 h of treatment. (**C**) The effect of FLU on STAT3 and cell cycle regulators expression. Cells were treated with 0.5 µM FLU or transfected with 50 nM siRNA against STAT3 and harvested after 48 h of treatment to determine the expression of cell cycle regulators. All western blot results were processed into single figure and original blots are available in supplementary files S2. *p ˂ 0.05 vs untreated control.

**Figure 4:**
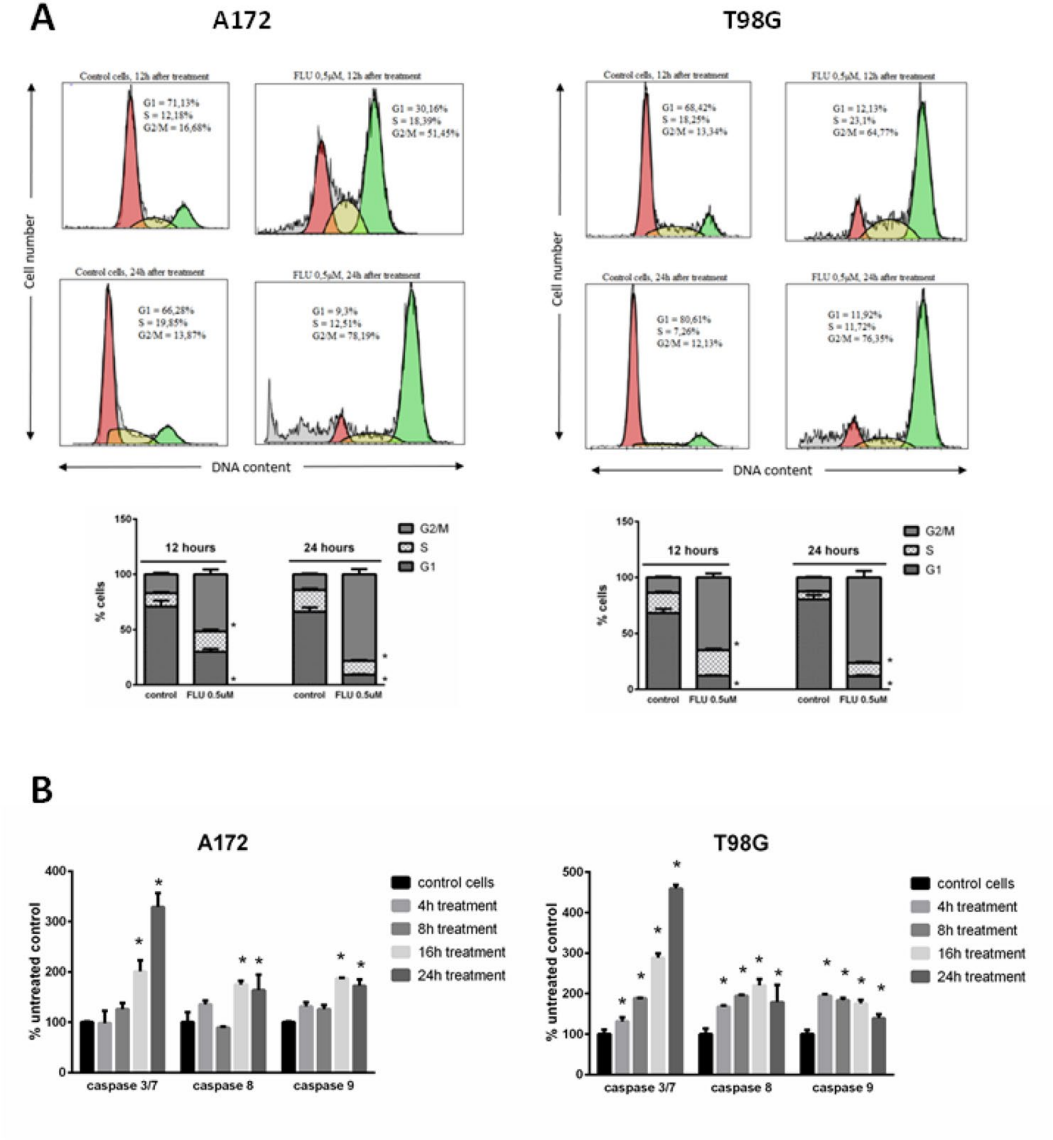
The effect of FLU on cell cycle distribution and caspase activation in A172 and T98G cells. (**A**) Cells were treated with 0.5 µM FLU and collected after 12 h and 24 h treatment to measure the cell cycle distribution. (**B**) Cell were treated with FLU 0.5 µM and the caspase activity was measured after 4 h, 8 h, 16 h, and 24 h of treatment. *p ˂ 0.05 vs untreated control.

Next, we examined the effect of FLU treatment on STAT3 expression and activation in A172 and T98G cell lines. First, we checked whether FLU exposure influences the expression of STAT3 and its activated form phospho-STAT3 (Tyr705) in various time intervals (24, 48 and 72 h of exposure) and upon several FLU concentrations (0.25 µM; 0.5 µM; 1 µM). Obtained results demonstrate that FLU mediated effect on STAT3 activity is not consistent and neither time nor concentration dependent in the employed GBM models. Conversely, FLU had a suppressive effect on the expression of STAT3, in particular in A172 cells, where a time and concentration dependence was visible too (Fig. 3A,B). Since these results suggested a measurable effect of FLU on STAT3 signaling pathway, in the next step we sought to mechanistically verify the functional relationship between STAT3 activation and p53 expression. Cells treated with FLU 0.5 µM were compared with cells transfected with siRNA against STAT3. Although FLU lowered the activation and expression of STAT3 in both used cell lines, increased expression of p53 and p21 could be observed only in A172 cells. Further, we observed the decrease in expression of Cdk1 and cyclin B1 in both tested cells lines, which suggested possible G2/M arrest of cells (Fig. 3C). Furthermore, partial effect on p53 expression in A172 and cyclin B1 expression in both cell lines was also observed in cells with silenced STAT3, confirming the connection between FLU and STAT3 activation/expression. To extend the validity of our findings and gain a deeper insight into the underlying mechanisms of FLU on GBM cells, cell cycle analysis via flow cytometry was performed. Already after 12 h of FLU treatment and more significantly after 24 h, the distribution of cell cycle phases showed marked changes compared to untreated control cells. After 24 h of treatment, majority of cells accumulated in G2/M phase (A172 78%, T98G 76%) with subsequent reduction of G1 (A172 9%, T98G, 12%) and S phase cells (A172 12%, T98G 12%) (Fig. 4A).

### FLU promoted caspase activation

Next, the activation of initiator caspases 8 and 9 and effector caspases 3/7 in GBM cells following the FLU 0.5 µM treatment was examined upon time intervals of 2 h, 4 h, 8 h and 24 h. In FLU-exposed cells, all examined caspases were activated in both tested cell lines, however not homogeneously. In A172 cells, initiator caspases 8 and 9 were activated between 8 and 24 h of FLU exposure while in T98G these caspases were active already at early time intervals. In both tested cell lines FLU increased the activation of caspases 3/7 in a time dependent manner with T98G cells showing again both signs of earlier activity as well as higher maximum values (Fig. 4B).

### FLU lowered STAT3 expression in GBM primary cultures

**Figure 5:**
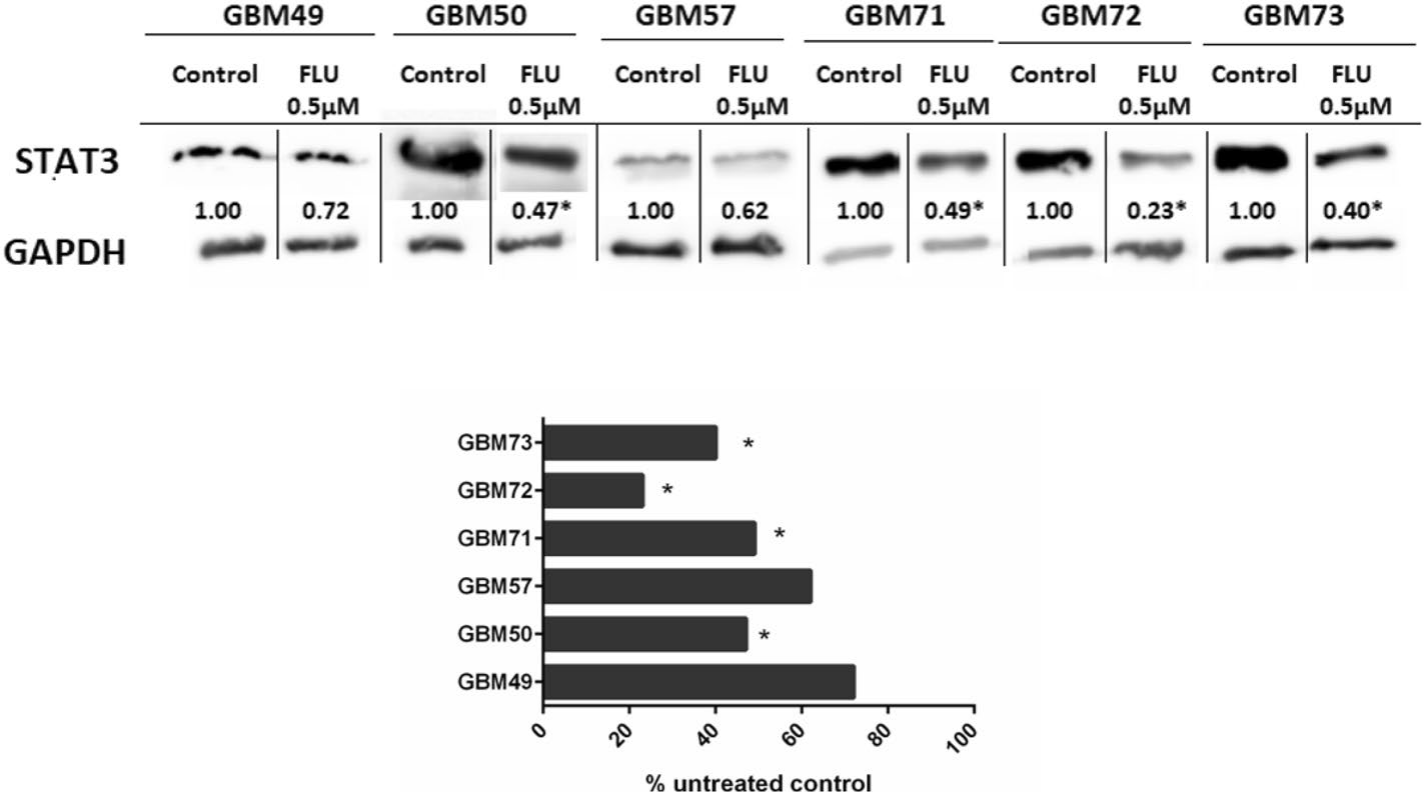
The STAT3 expression changes after FLU treatment in GBM primary cultures. Cells were treated with FLU 0.5 µM for 48 h and the relative quantification of proteins was measured using western blot using GAPDH as loading control. Changes in expression were expressed as percentage of untreated control. *p ˂ 0.05 vs untreated control.

In view of the FLU effect on STAT3 expression in A172 an T98G cell lines, next set of experiments was performed on GBM primary cultures. In all our tested primary cultures, FLU lowered the STAT3 expression and in GBM57, GBM71, GBM72 and GBM73 cultures this decrease was statistically significant (Fig. 5).

### STAT3 clinical relevance

**Figure 6:**
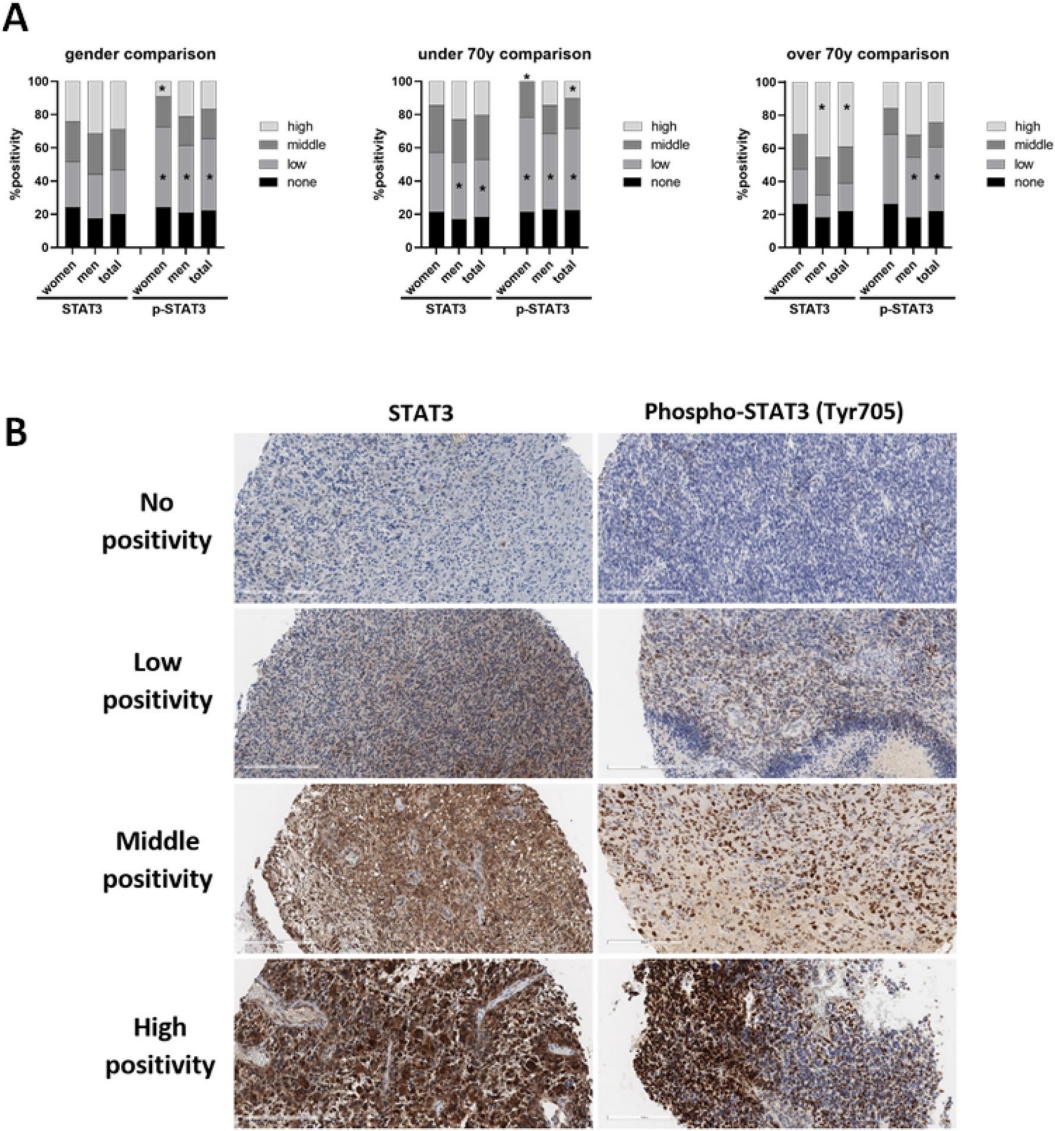
Immunohistochemical analysis of STAT3 and phospho-STAT3 expression in clinical samples. (**A**) Quantification of analysis of STAT3 and phospho-STAT3 expression in clinical samples. (**B**) Representative immunohistochemical images of groups with none, low, middle and high positivity in both STAT3 and phospho-STAT3 expression. Blue stained cells represent negative population, brown stained cells represent positive population. Magnification 200x, scale bar 200 µm. *p ˂ 0.05 significant difference in expression of STAT3, or p-STAT3 vs none positivity.

The immunohistochemical analysis of STAT-3 and p-STAT3 was carried out using 90 clinical samples, evenly distributed by the clinically relevant age limit of 70 years. Our results demonstrated the presence of at least low expression of STAT3 and p-STAT3 in more than 75% of examined cases, with high expression being detected in 29%, resp. 17% of all examined cases (Fig. 6). In patients over 70 years of age, high expression of STAT3 was recorded in 39% of cases and in over 45% cases of male gender in this group.

## Discussion

Current major limitations of GBM chemotherapy include restricted entry of candidate drugs to the brain via blood–brain barrier as well as innate and developed chemoresistance of malignant cells. Both of these disadvantages do not seem apply to FLU as well as other benzimidazoles as these accumulate in the brain and are not a subject of known drug efflux systems^27^. Thus FLU was previously demonstrated to be effective against a range of solid tumors and leukemic cells^15,16,28^ as well as glioma cells^17^. In the extensive study of Ren et al., FLU was also identified to have anti-GBM effect among other benzimidazoles^18^. In our work, we confirmed the antiproliferative effect of FLU not only on standard GBM cell lines but also on primary GBM cultures derived from patient’s samples. The inhibitory efficacy of FLU was generally significantly higher compared to reference GBM agent TMZ (the lowest IC50 FLU 0.6 µM versus the lowest IC50 TMZ 990 µM). Use of real-time cell analysis also revealed distinct duration and extent of FLU-dependent inhibitory effect. In stabilized GBM cell lines A172 and T98G FLU-mediated inhibition was robust and stable over 72 h treatment time whereas proliferation of FLU-exposed primary GBM cultures (GBM49 and GBM72) despite their initial inhibition was gradually renewed albeit in a slower manner. Varying cell sensitivity to either FLU or TMZ treatment in this case may be explained by a number of reasons including generally heterogenous character of GBM cell lines^2^, biological differences of distinct cell models^26^ as well as the range of pharmacodynamic parameters such as the expression of drug targets. In this context it is known that sensitivity of GBM cells to classical microtubule addressing agents (i.e. taxanes) tightly correlates with baseline levels of α/β tubulin but not with tubulin isotypes and their posttranslational modifications^29^. We have not directly measured and compared these α/β tubulin levels in our model cells prior exposure to FLU although different expression of individual tubulins might have been present given the unique nature of individual used GBM cells. Whether this varying tubulin expression matches with sensitivity of particular GBM cells to FLU in a manner similar to other microtubule inhibitors is an intriguing avenue which will be investigated in future.

Benzimidazoles including FLU were previously reported to exert their effect by binding to β-tubulin and inhibiting microtubule function and structure^14,30^ and our results agree with these reports. Furthermore, they extend them by showing that FLU also influences to the different degree α- and βIII-tubulin expression. FLU-treated cells changed the content and organization of microtubular network which externally associated with altered cell shape and size, often leading to the appearance of large multinucleated cells. Cycling of treated cells was generally disrupted, cells accumulated in G2/M phase while increasing expression of cell cycle regulators p53 and p21 similarly to previous reports^17^. This combined evidence suggests that FLU induced mitotic catastrophe associated with cell cycle arrest as an often connected cell phenotype with the use of microtubule targeting agents^31^. Mitotic catastrophe is a suppressive mechanism that promotes elimination of aberrantly dividing cells by triggering the cell-death signaling^31^. The common outcome of this process is thus cell death—apoptosis or necrosis. Behavior of FLU-exposed GBM cells, namely their loss of attachment, characteristic rounding with membrane blebbing and increased activity of initiator and effector caspases clearly demonstrated the presence of caspase-induced cell death.

While it is already known that FLU targets microtubules and inhibits vital life features of many different cancer cell types^19^, the exact mechanism or sequence of events underlying it remain unclear. Recently, several studies reported STAT3 and its activation as another important target of FLU in cancer cells^20,21^. STAT3 is nowadays regarded a key molecule integrating several major oncogenic signaling pathways including the one driving mesenchymal transformation^32^. The expression of STAT3 and its activation in GBMs varies considerably, being estimated between 9 and 83% in retrospective studies^24^. In our examined cohort of GBM patient’s tissue samples, STAT3 expression and activation was present in over 75% of cases, with 40% of patients over 70 years age showing high STAT3 expression. Similarly heterogeneous presence of STAT3 was noted in the used stabilized GBM cell lines and primary GBM explants. Their treatment with FLU inhibited STAT3 activation and even STAT3 expression in a concentration dependent manner and induced the signaling pathway leading to G2/M arrest. This effect could be also observed in cells with a reduced STAT3 expression, confirming the importance of STAT3 as a target and mediator of FLU. In addition, the effect of FLU on STAT3 expression was shown in all employed models with significance seemingly copying the effect of FLU on βIII-tubulin expression. Although STAT3 is believed to be located in cytoplasmic pool in cell^33^, other studies suggest its possible association with microtubules^34^, thereby suggesting another interesting line of possible future research.

## Conclusion

In our study, FLU demonstrated its inhibitory effects towards all employed GBM models, proving to be more effective than currently used TMZ. Causing observable morphological changes and disruption of microtubule networks with concomitant multinucleation, FLU effects were further associated with suppression of STAT3 expression and activity and cell cycle arrest at G2/M phase leading to caspase-dependent cell death.

## Supplementary Information

Supplementary Information 1.

Supplementary Information 2.

## Data Availability

The dataset generated and analyzed during the current study are available from corresponding author on reasonable request.
