# Author Correction: Flubendazole exhibits anti-glioblastoma effect by inhibiting STAT3 and promoting cell cycle arrest

**DOI:** 10.1038/s41598-023-37369-6

**Published:** 2023-06-23

**Authors:** Barbora Vítovcová, Veronika Skarková, Radim Havelek, Jiří Soukup, Ananya Pande, Kateřina Caltová, Emil Rudolf

**Affiliations:** 1 Department of Medical Biology and Genetics, Faculty of Medicine in Hradec Králové, Charles University, Šimkova 870, 500 03 Hradec Králové, Czech Republic; 2 Department of Medical Biochemistry, Faculty of Medicine in Hradec Králové, Charles University, Šimkova 870, 500 03 Hradec Králové, Czech Republic; 3 The Fingerland Department of Pathology, Faculty of Medicine and University Hospital in Hradec Králové, Charles University, Sokolská 581, 500 05 Hradec Králové, Czech Republic

Correction to: *Scientific Reports* 10.1038/s41598-023-33047-9, published online 12 April 2023

The Acknowledgements section in the original version of this Article was incomplete. It now reads:

This study was supported by the Ministry of Health, Czech Republic, project No. NU20-03-00360. We are grateful to Dr. Petr Krupa and Dr. Michael Bartos from Department of Neurosurgery, Faculty Hospital in Hradec Kralove, for providing GBM source samples.

The original Article has been corrected.

