# Supplementary material for: Flubendazole exhibits anti-glioblastoma effect by inhibiting STAT3 and promoting cell cycle arrest
Source: Sci Rep. 2023 Apr 12;13:5993. doi: 10.1038/s41598-023-33047-9 (PMC10097688; doi:10.1038/s41598-023-33047-9)
Supplement: Supplementary file 1 — Supplementary Information 1. [file 41598_2023_33047_MOESM1_ESM.pdf]

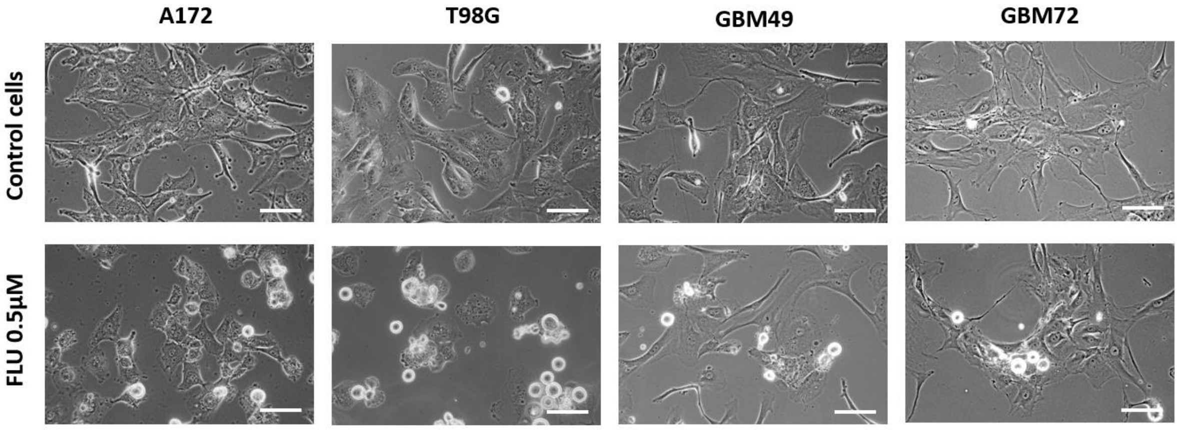

Fig. S1. The effect of FLU on proliferation of GBM cells after 24h treatment. Morphological changes and multinucleation caused by FLU 0.5 μM in GBM cells. Cells were treated accordingly and observed by phase contrast microscopy during up to 72 h, here represented by images acquired in 24h time point. Magnification 400x, scale bar 20 μm.

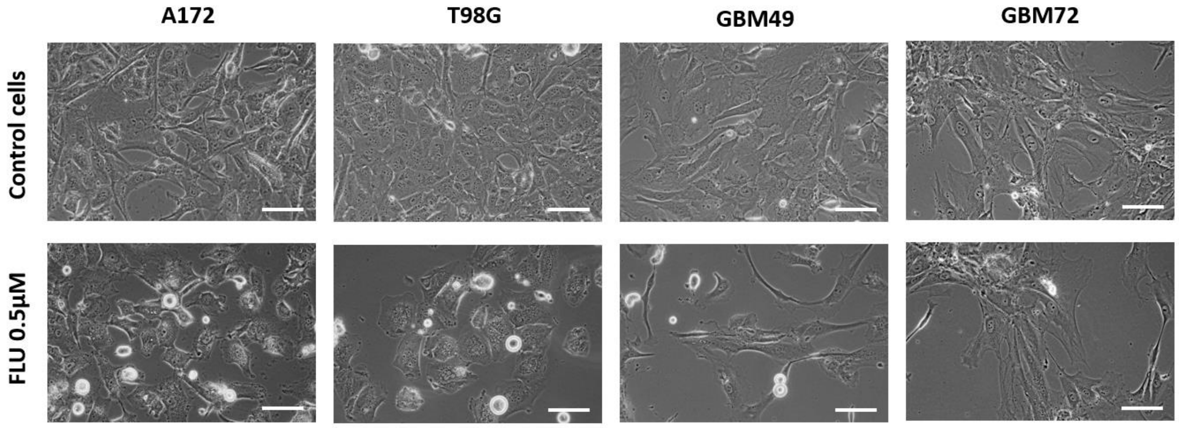

Fig. S2. The effect of FLU on proliferation of GBM cells after 48h treatment. Morphological changes and multinucleation caused by FLU 0.5 μM observed using phase contrast microscopy, shown images represent the 48 h time point. Magnification 400x, scale bar 20 μm.

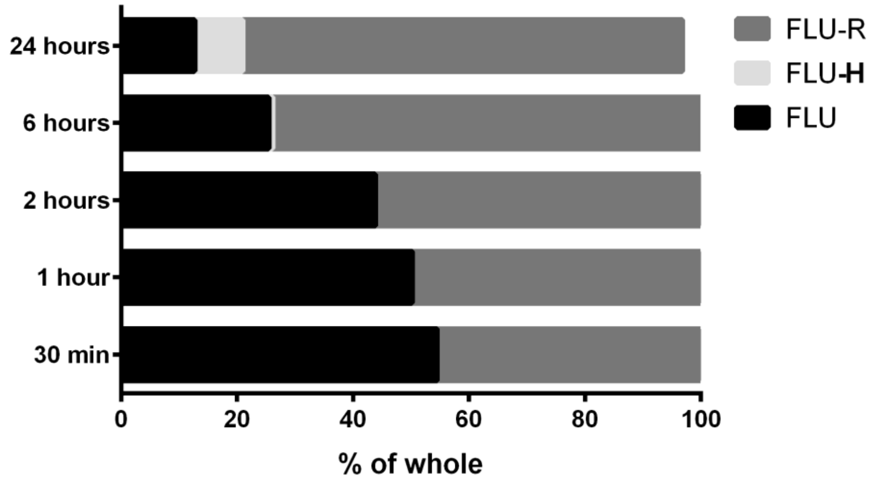

Fig. S3. The FLU entry into the brain of nude mice. Tested organism were injected with FLU in concentration of 25mg/kg and the levels of FLU and its two main metabolites reduced-FLU (FLU-R) and hydroxy-FLU (FLU-H) were measured 30 min, 1 h, 2 h, 6 h and 24 h after administration.

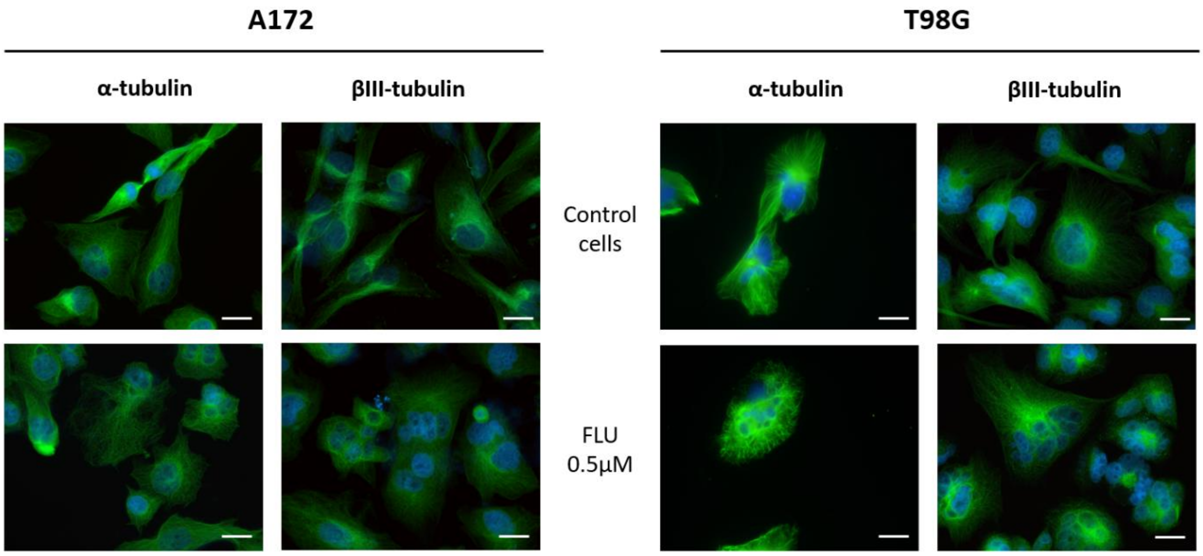

Fig. S4. The effect of FLU on α- and βIII-tubulin in GBM cells treated with 0.5 µM FLU for 24h. The changes in α- and βIII-tubulin in A172 and T98G observed via fluorescence microscopy (magnification 600x, scale bar 10 µm).

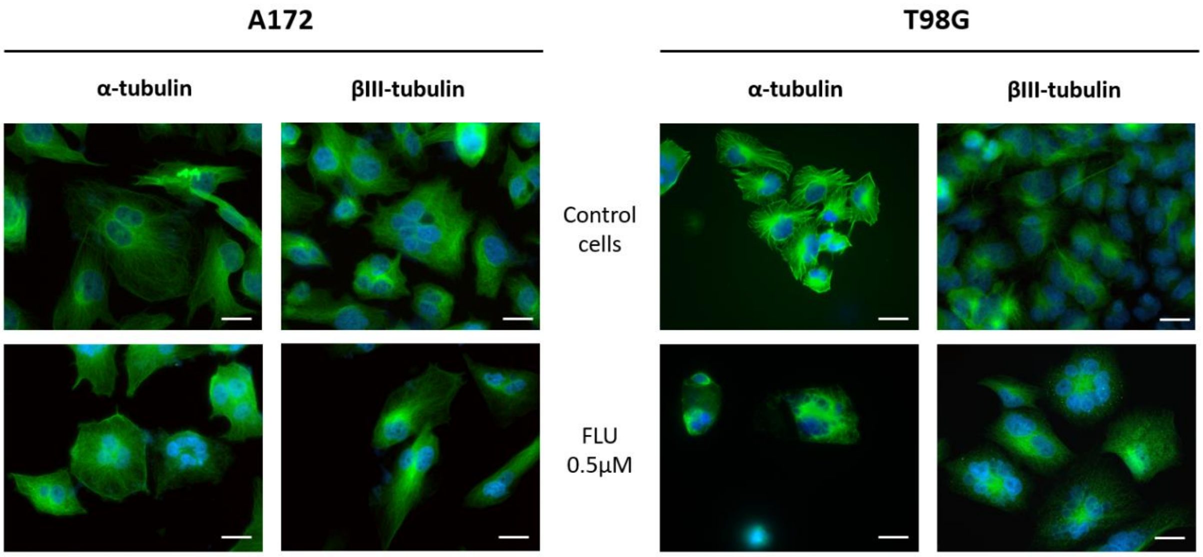

Fig. S5. The effect of FLU on α- and βIII-tubulin in GBM cells treated with 0.5 µM FLU for 72h. The changes in α- and βIII-tubulin in A172 and T98G observed via fluorescence microscopy (magnification 600x, scale bar 10 µm)
