# Supplementary material for: Flubendazole exhibits anti-glioblastoma effect by inhibiting STAT3 and promoting cell cycle arrest
Source: Sci Rep. 2023 Apr 12;13:5993. doi: 10.1038/s41598-023-33047-9 (PMC10097688; doi:10.1038/s41598-023-33047-9)

# Supplementary material – original western blot files

## Original western blot files for figures 2B and 3A – A172

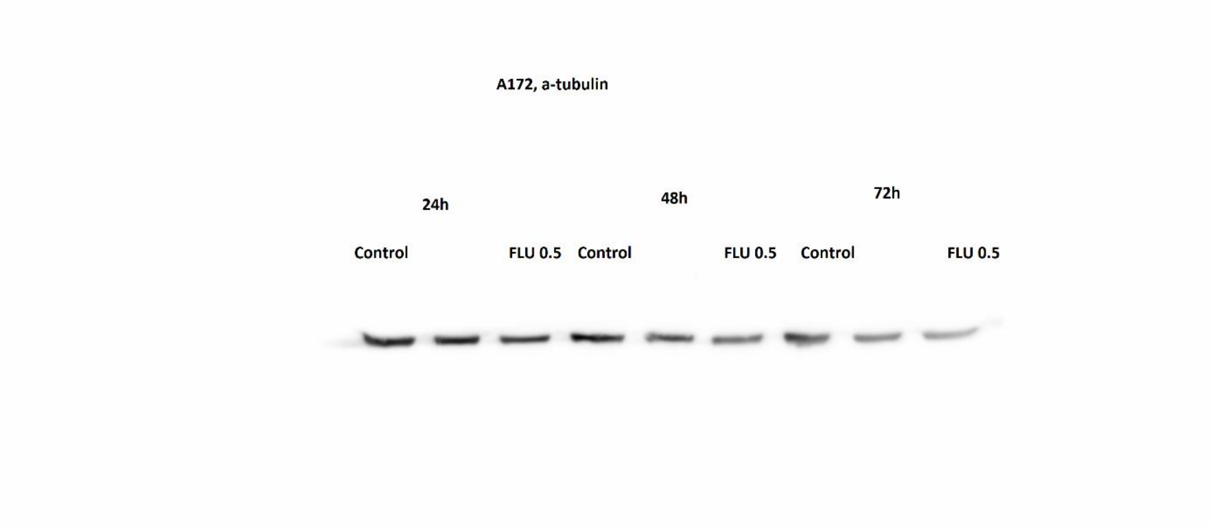


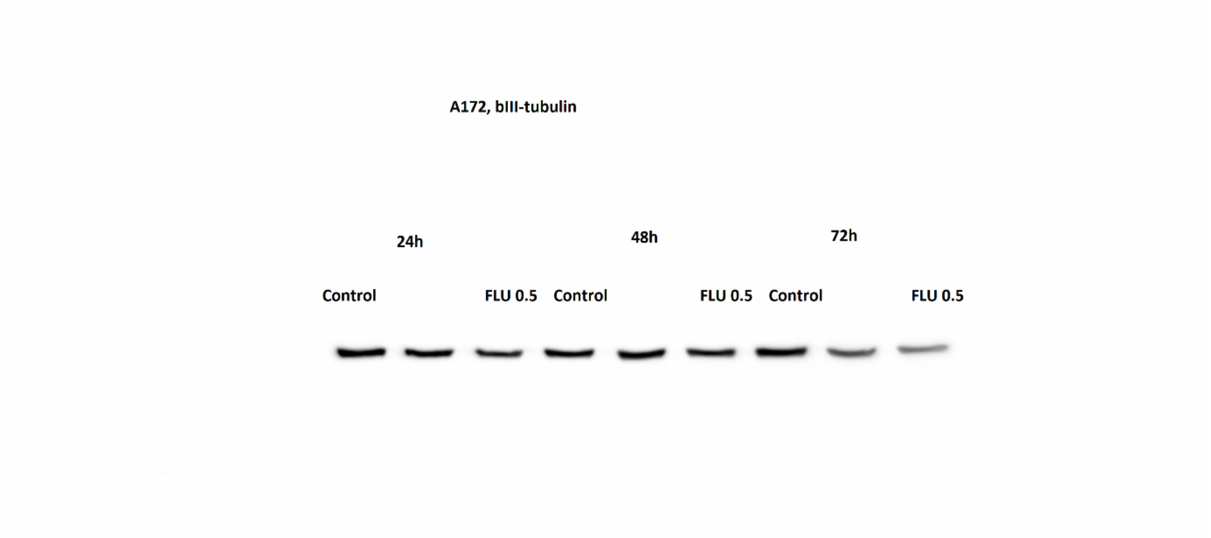


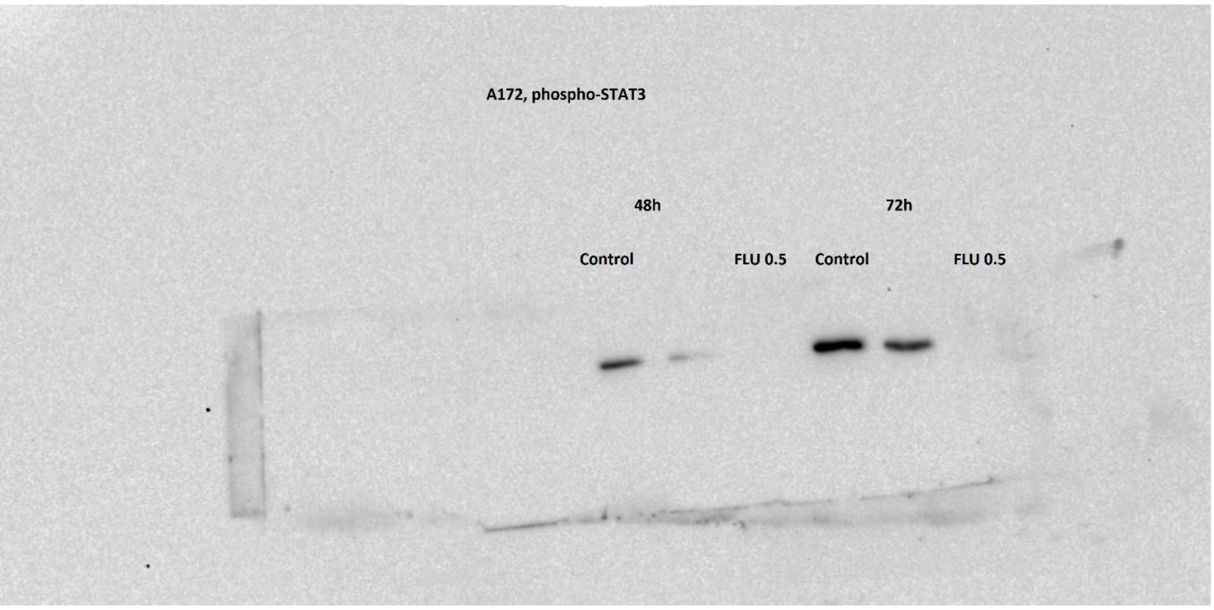

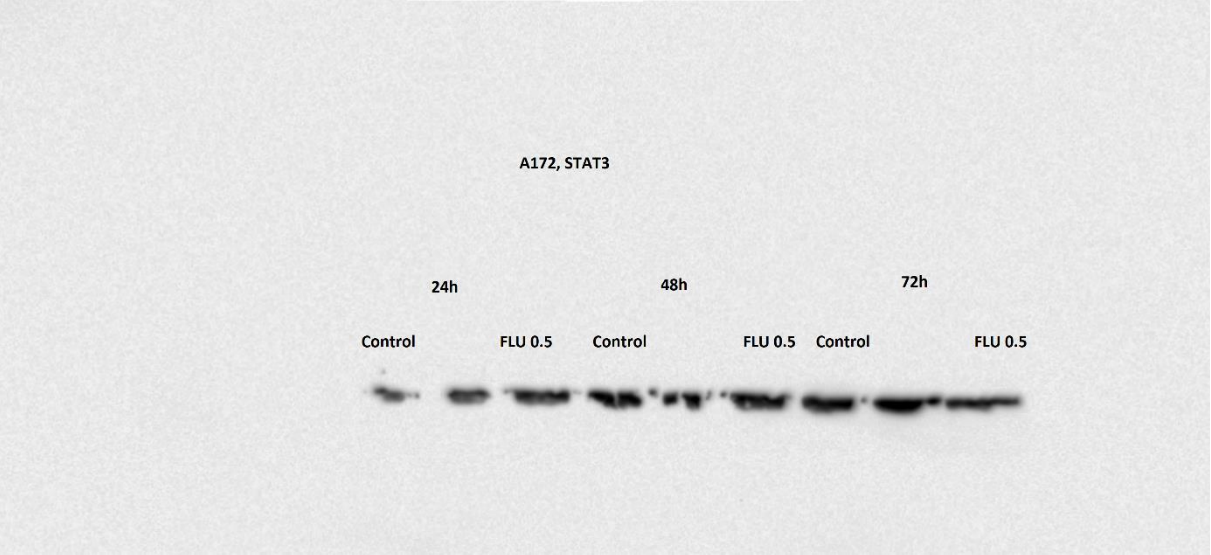
A172, STAT3
24h
48h
72h
Control
FLU 0.5
Control
FLU 0.5
Control
FLU 0.5

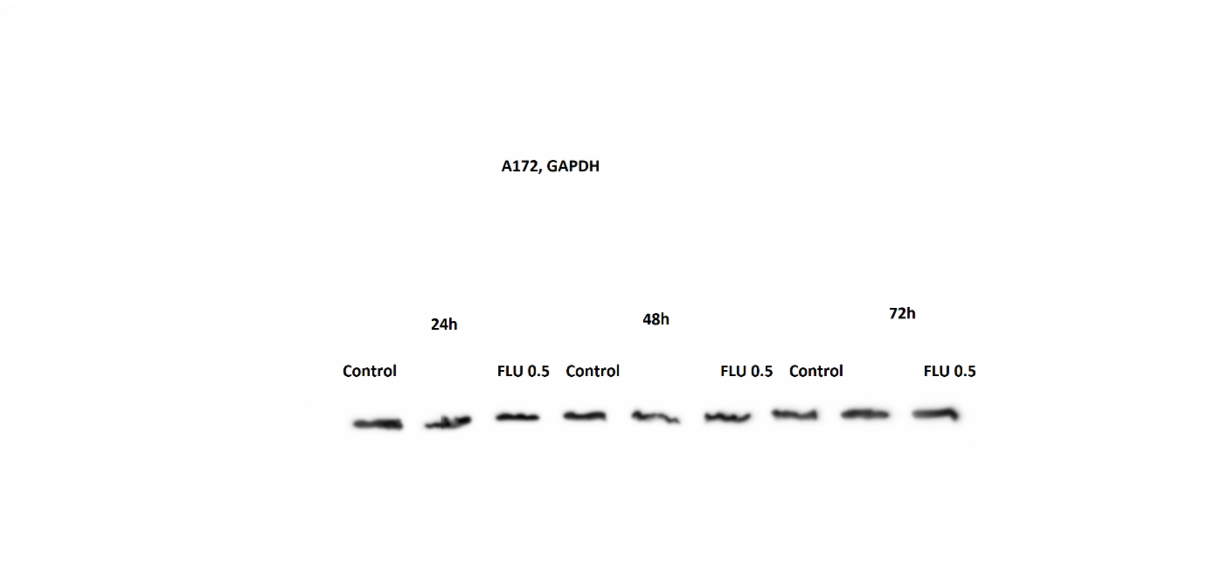
A172, GAPDH
24h
48h
72h
Control
FLU 0.5
Control
FLU 0.5
Control
FLU 0.5

A172, a-tubulin

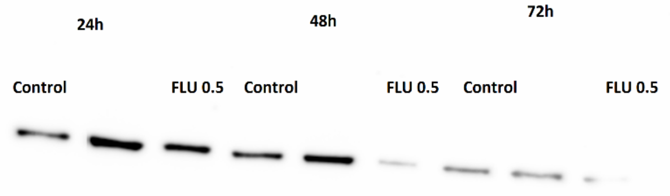


A172, bIII-tubulin

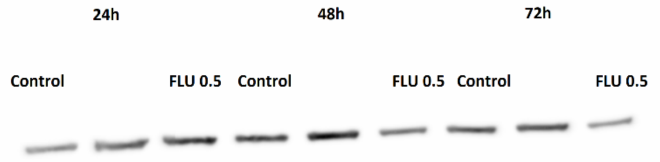

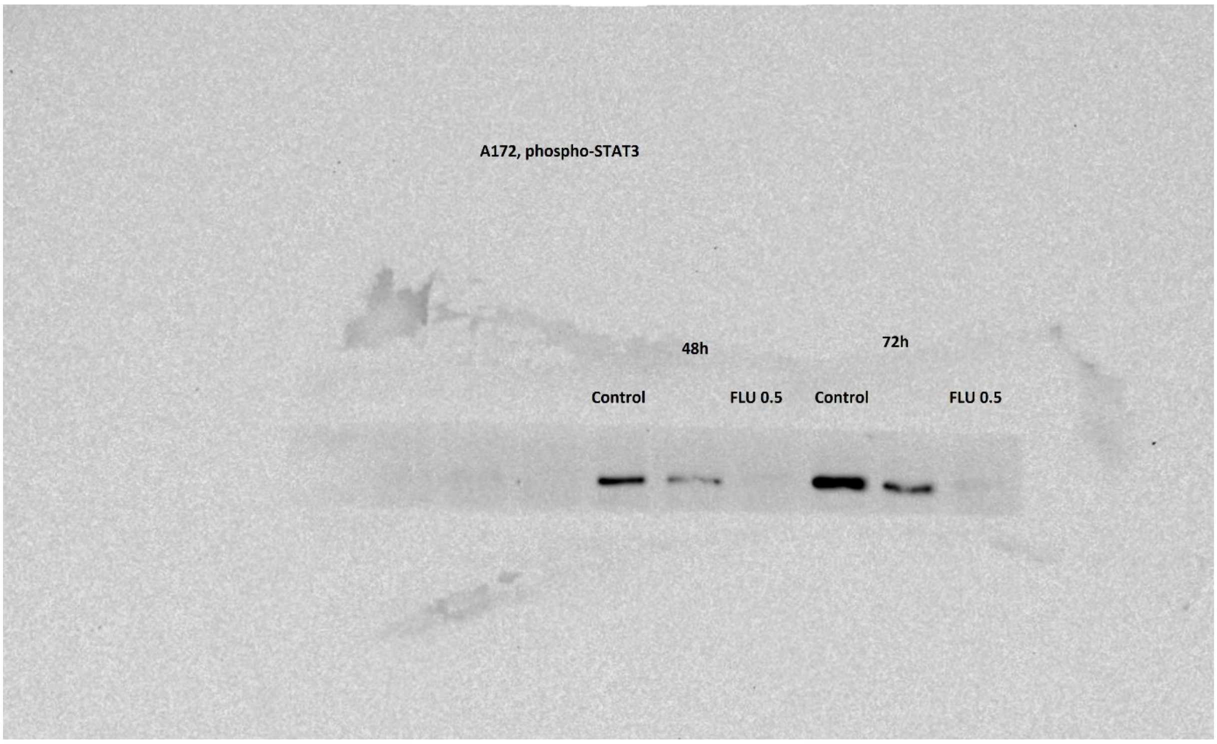
A172, phospho-STAT3
48h
72h
Control
FLU 0.5
Control
FLU 0.5

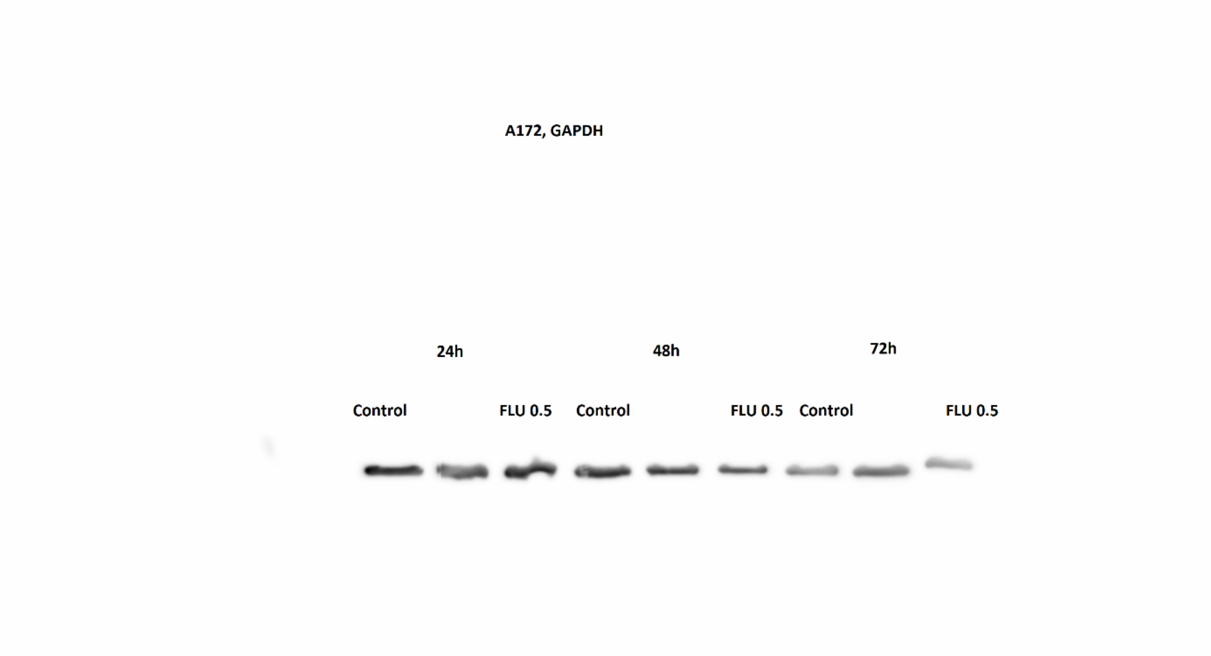
A172, GAPDH
24h
48h
72h
Control
FLU 0.5
Control
FLU 0.5
Control
FLU 0.5

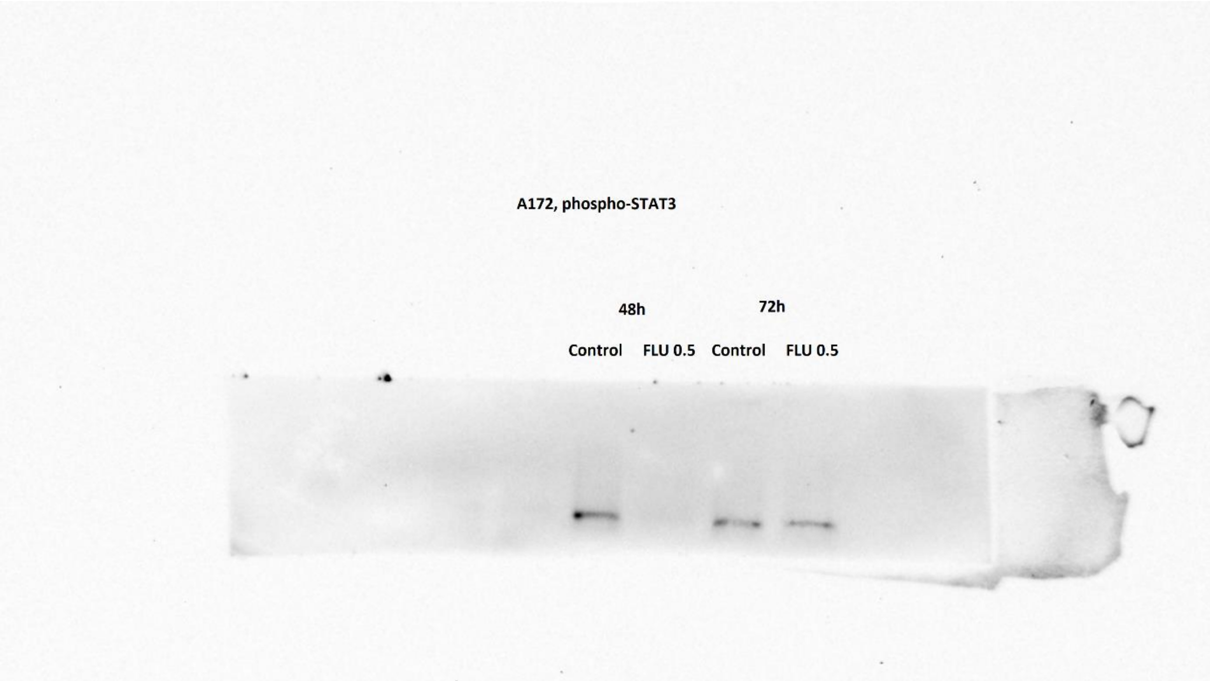
A172, phospho-STAT3
48h
72h
Control
FLU 0.5
Control
FLU 0.5

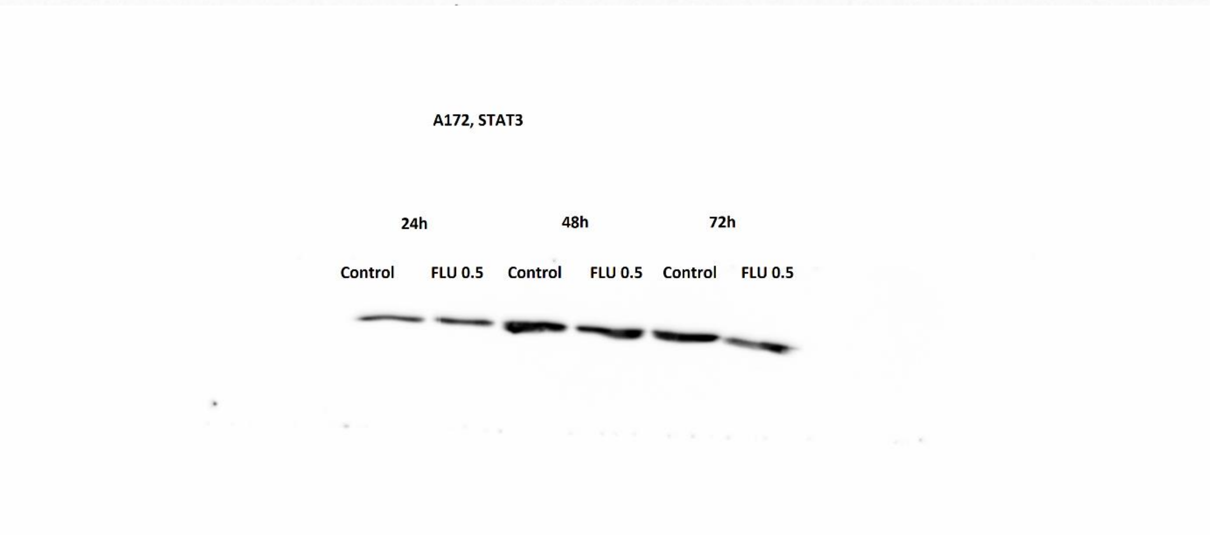
A172, STAT3
24h
48h
72h
Control
FLU 0.5
Control
FLU 0.5
Control
FLU 0.5

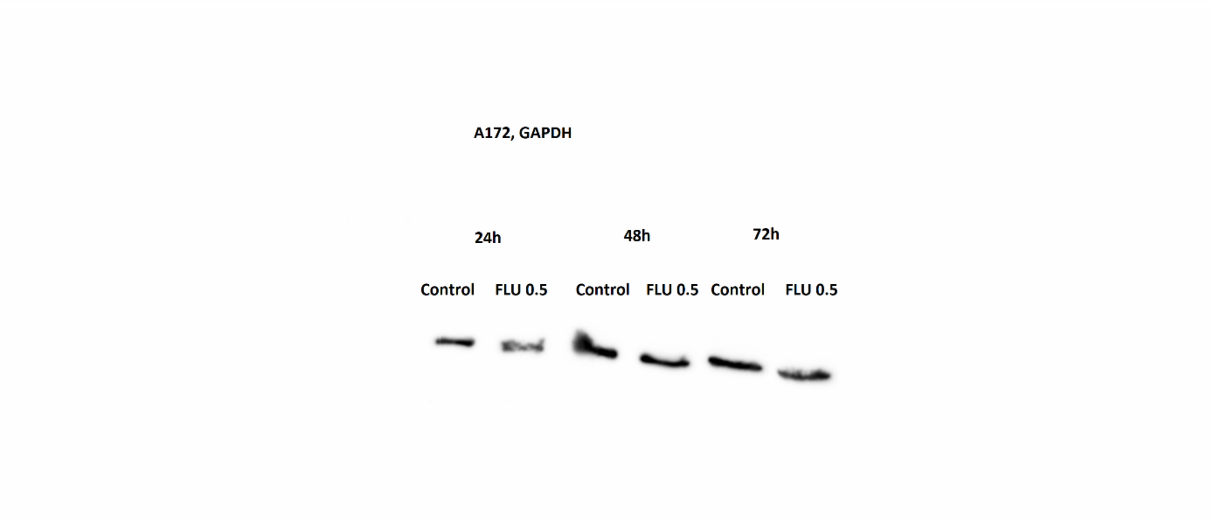
A172, GAPDH
24h
48h
72h
Control
FLU 0.5
Control
FLU 0.5
Control
FLU 0.5

**Original western blot files for figures 2B and 3A – T98G**

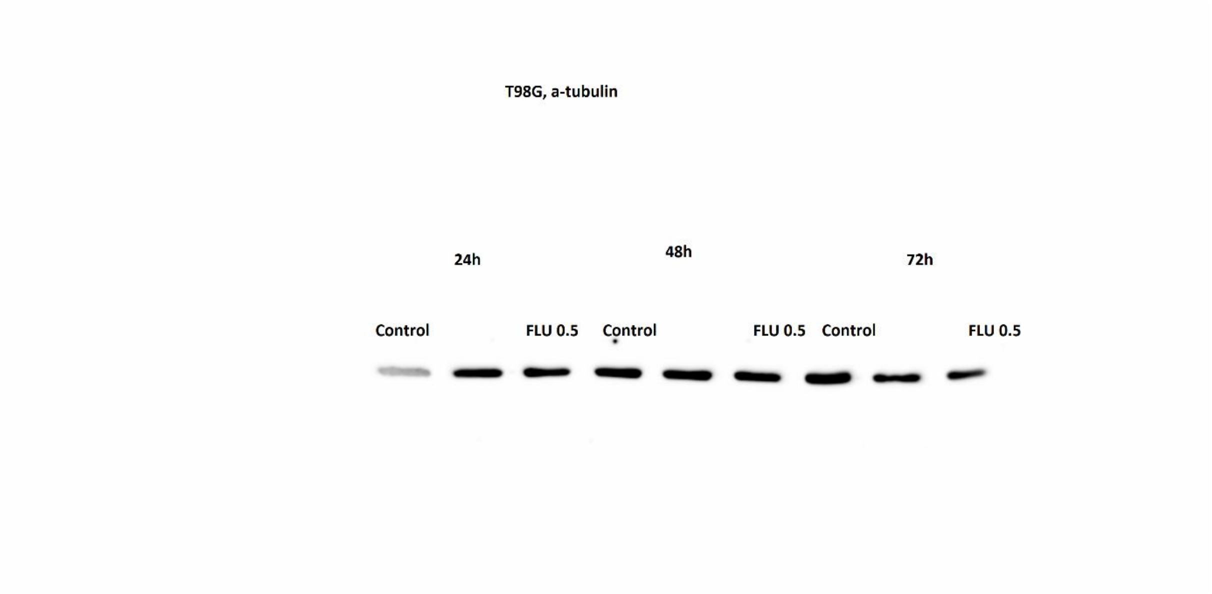


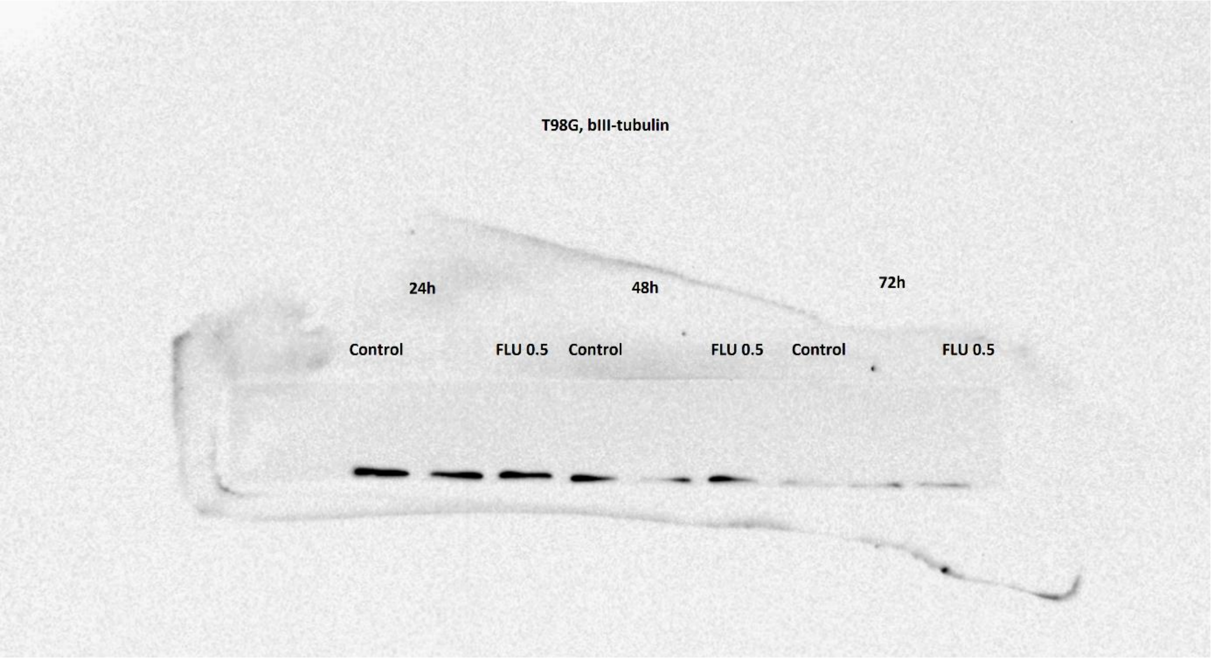

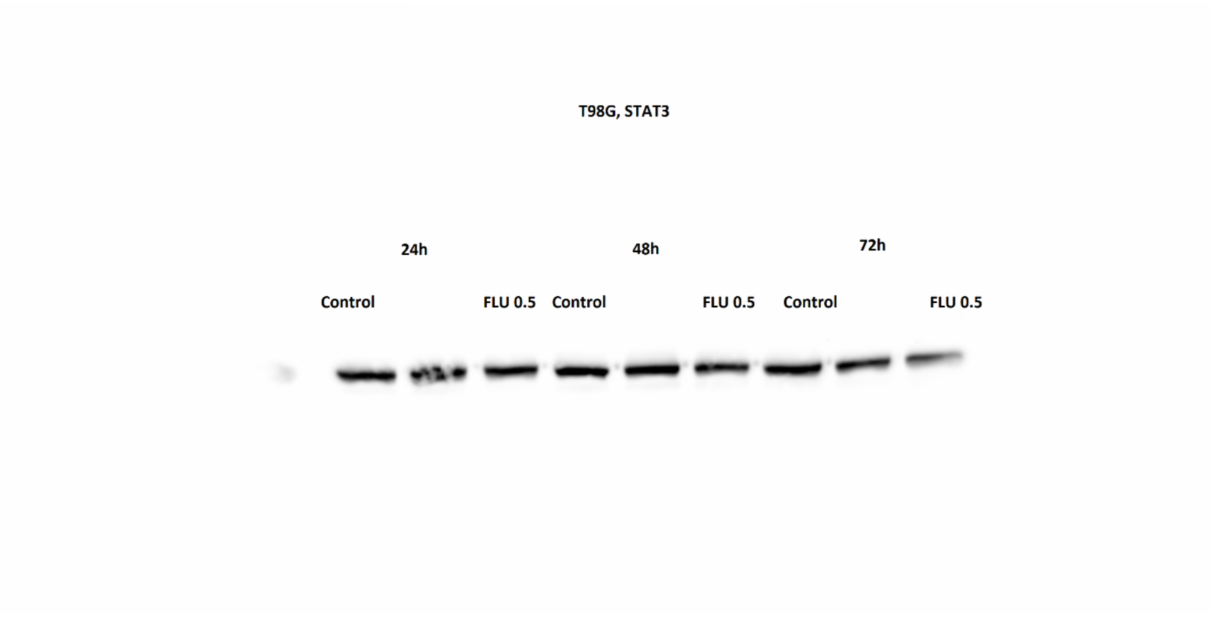
T98G, STAT3
24h
48h
72h
Control
FLU 0.5
Control
FLU 0.5
Control
FLU 0.5

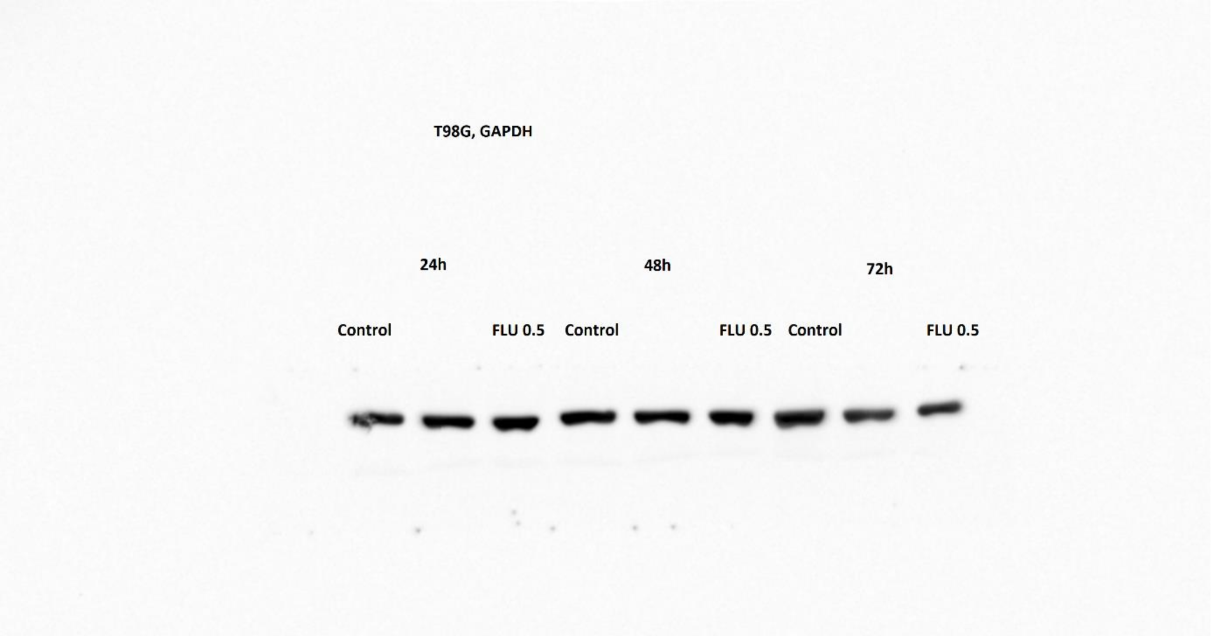
T98G, GAPDH
24h
48h
72h
Control
FLU 0.5
Control
FLU 0.5
Control
FLU 0.5

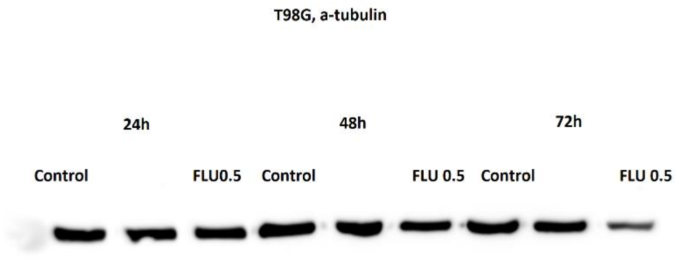
T98G, a-tubulin
24h
48h
72h
Control
FLU0.5
Control
FLU 0.5
Control
FLU 0.5

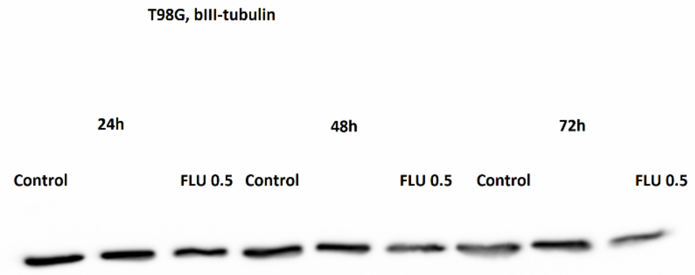
T98G, bIII-tubulin
24h
48h
72h
Control
FLU 0.5
Control
FLU 0.5
Control
FLU 0.5

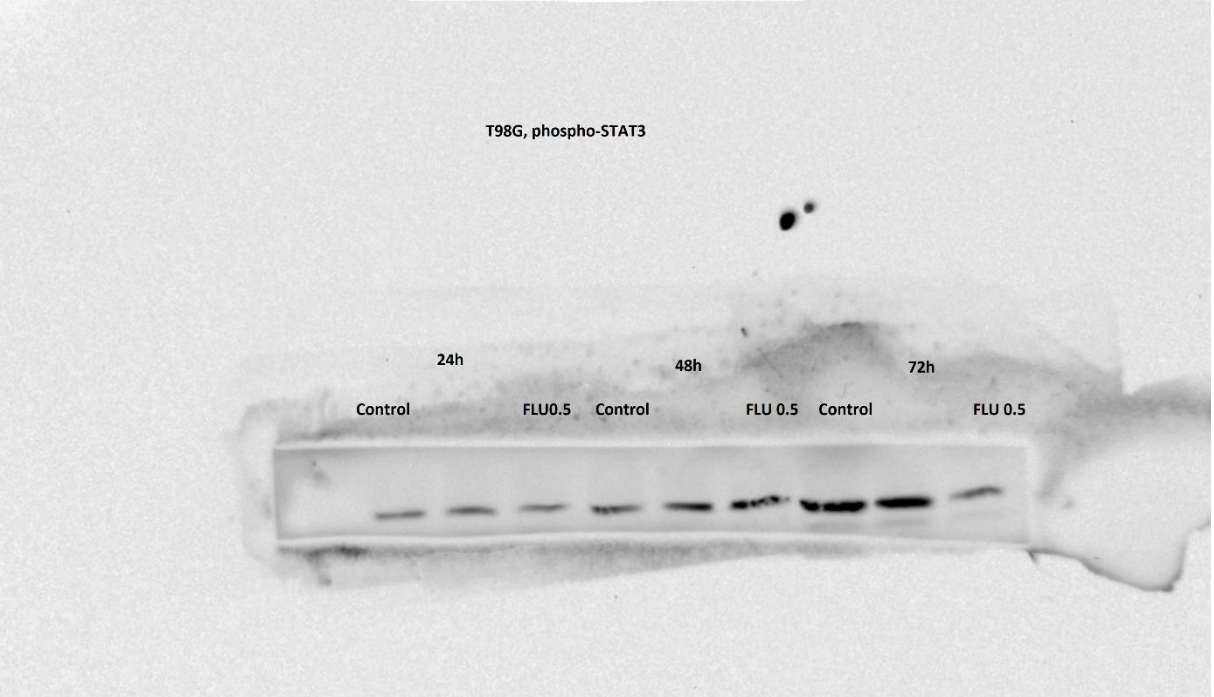
T98G, phospho-STAT3
24h
48h
72h
Control
FLU0.5
Control
FLU 0.5
Control
FLU 0.5

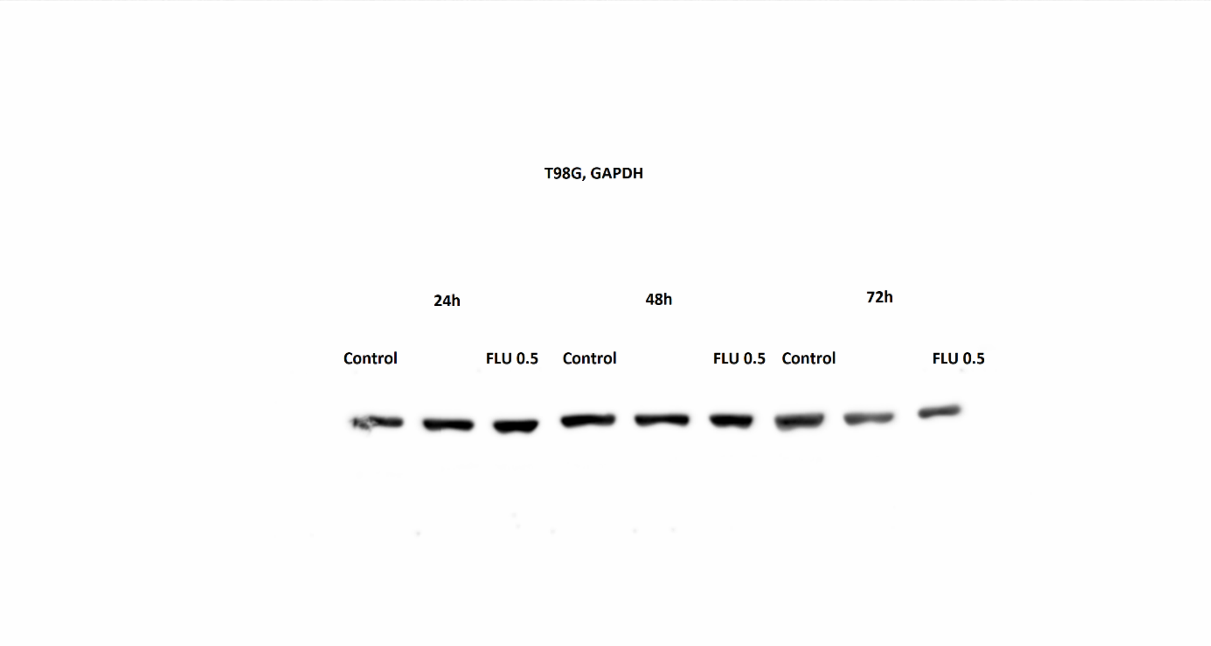
T98G, GAPDH
24h
48h
72h
Control
FLU 0.5
Control
FLU 0.5
Control
FLU 0.5

T98G, phospho-STAT3

24h 48h 72h

Control FLU 0.5 Control FLU 0.5 Control FLU 0.5

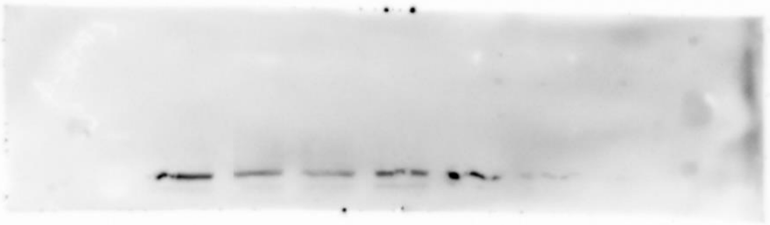

T98G, STAT3

24h 48h 72h

Control FLU 0.5 Control FLU 0.5 Control FLU 0.5

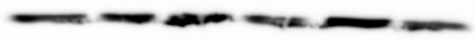

T98G, GAPDH

24h 48h 72h

Control FLU 0.5 Control FLU 0.5 Control FLU 0.5

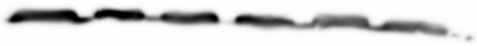

# Original western blot files for figure 3A

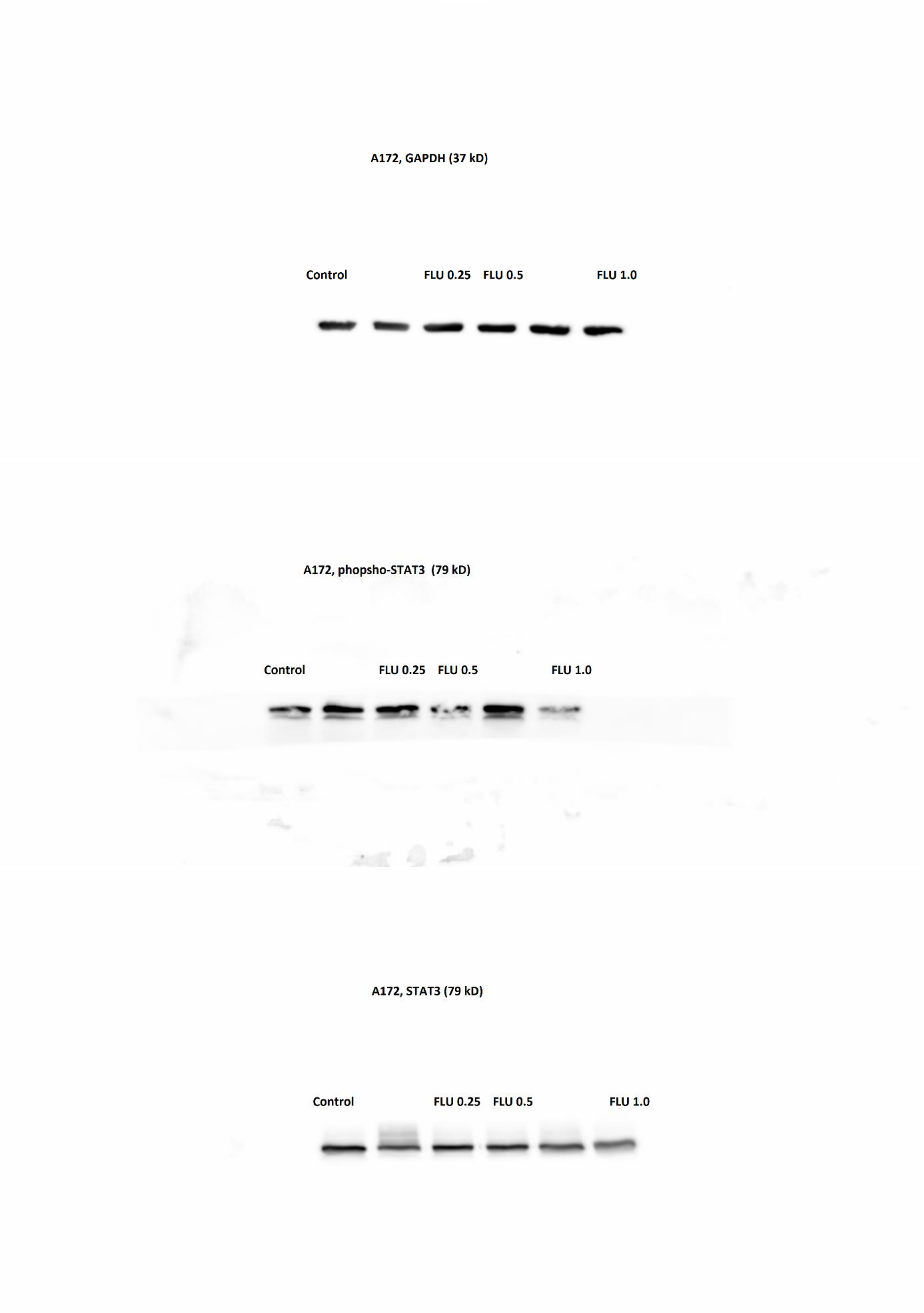

A172, GAPDH (37kD)

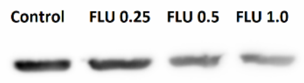


A172, phospho-STAT3 (79 kD)

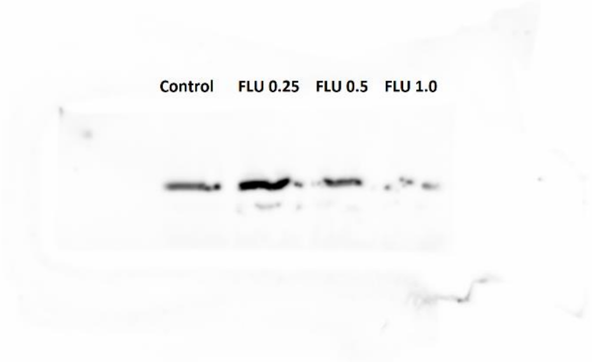


A172, STAT3 (79 kD)

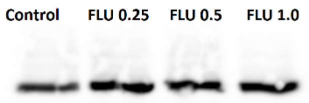

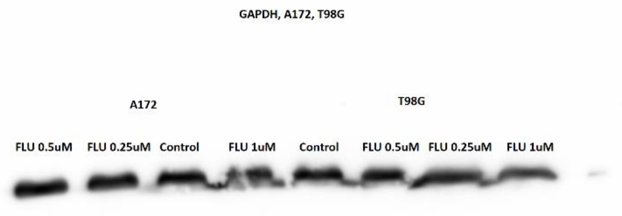
GAPDH, A172, T98G
A172
T98G
FLU 0.5uM
FLU 0.25uM
Control
FLU 1uM
Control
FLU 0.5uM
FLU 0.25uM
FLU 1uM

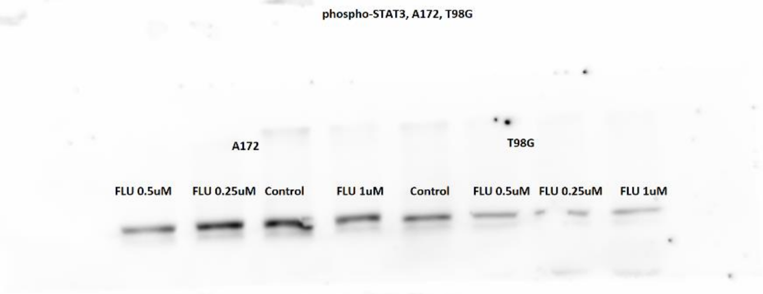
phospho-STAT3, A172, T98G
A172
T98G
FLU 0.5uM
FLU 0.25uM
Control
FLU 1uM
Control
FLU 0.5uM
FLU 0.25uM
FLU 1uM

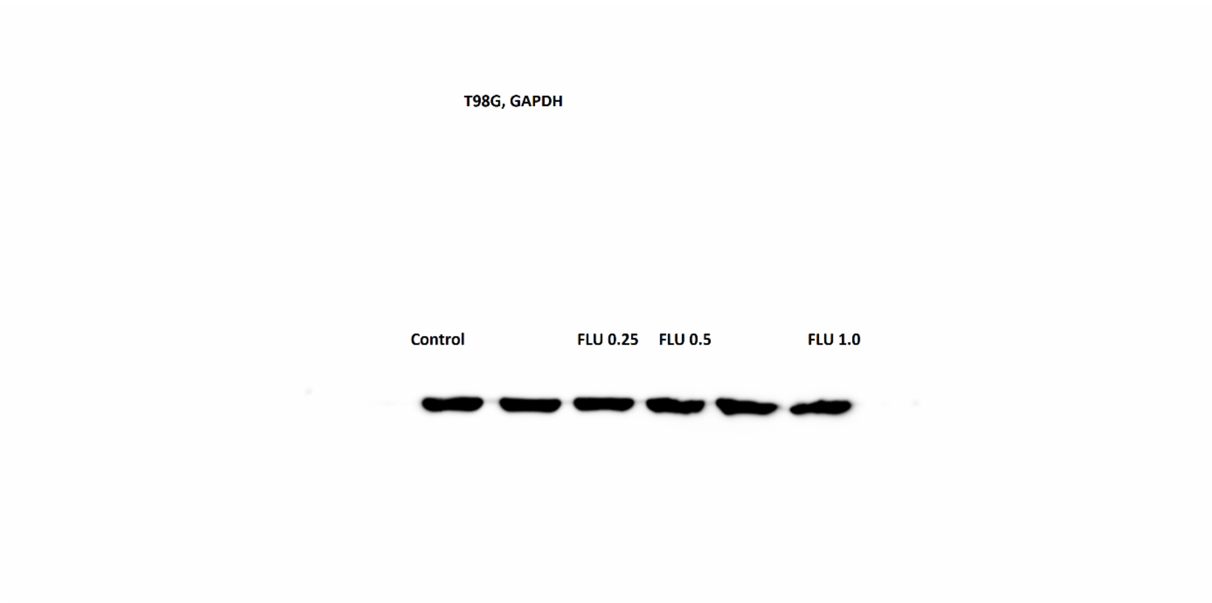
T98G, GAPDH
Control
FLU 0.25
FLU 0.5
FLU 1.0

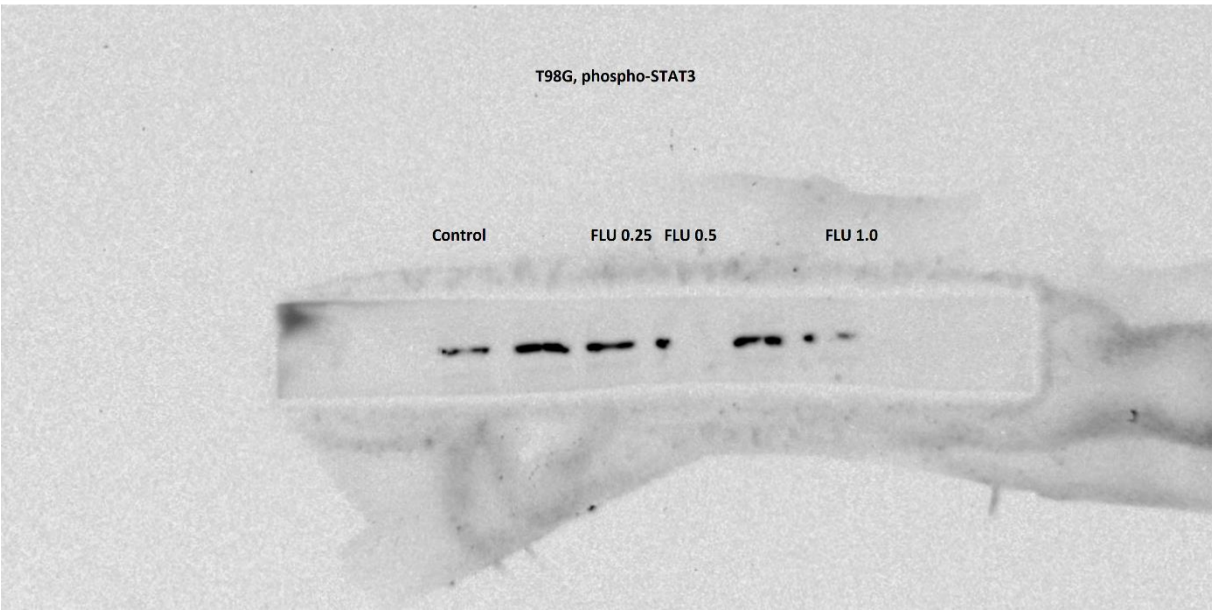
T98G, phospho-STAT3
Control
FLU 0.25
FLU 0.5
FLU 1.0

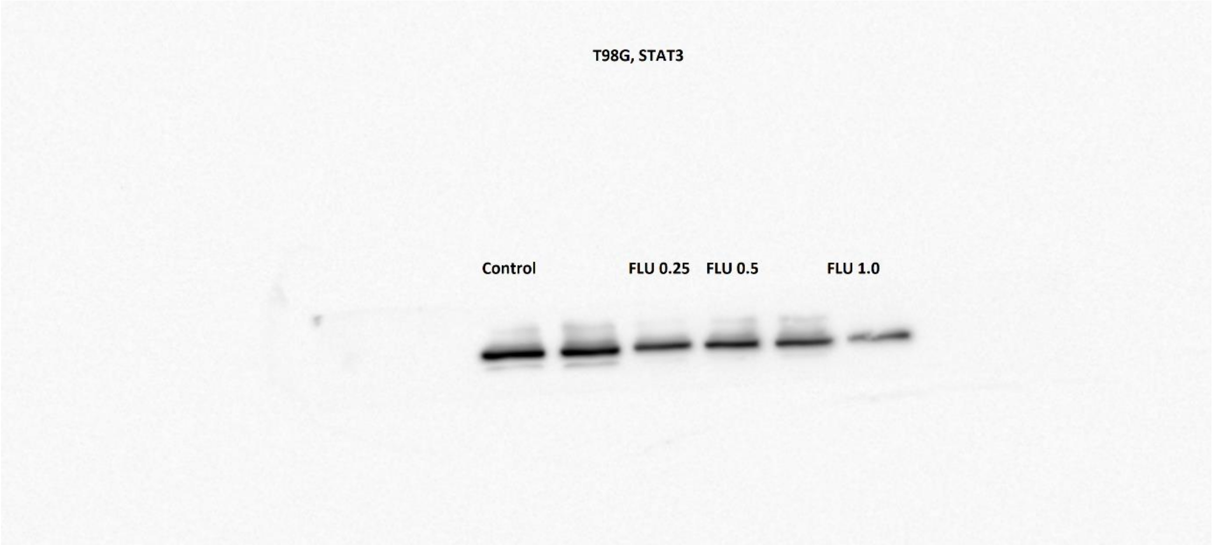
T98G, STAT3
Control
FLU 0.25
FLU 0.5
FLU 1.0

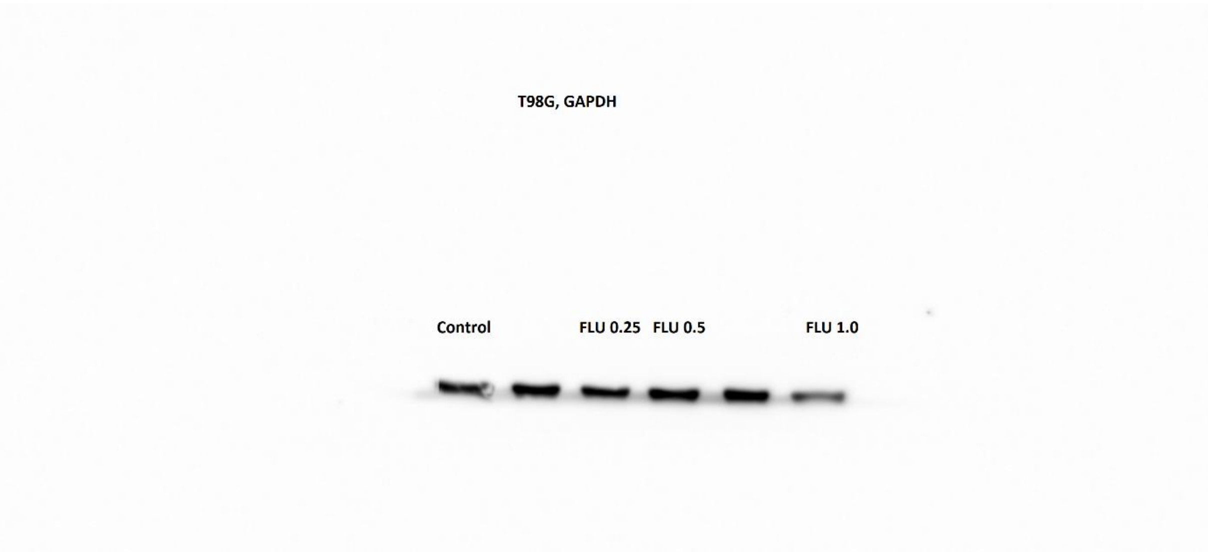
T98G, GAPDH
Control
FLU 0.25
FLU 0.5
FLU 1.0

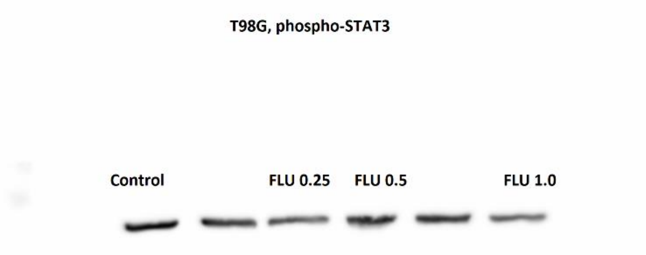
T98G, phospho-STAT3
Control
FLU 0.25
FLU 0.5
FLU 1.0

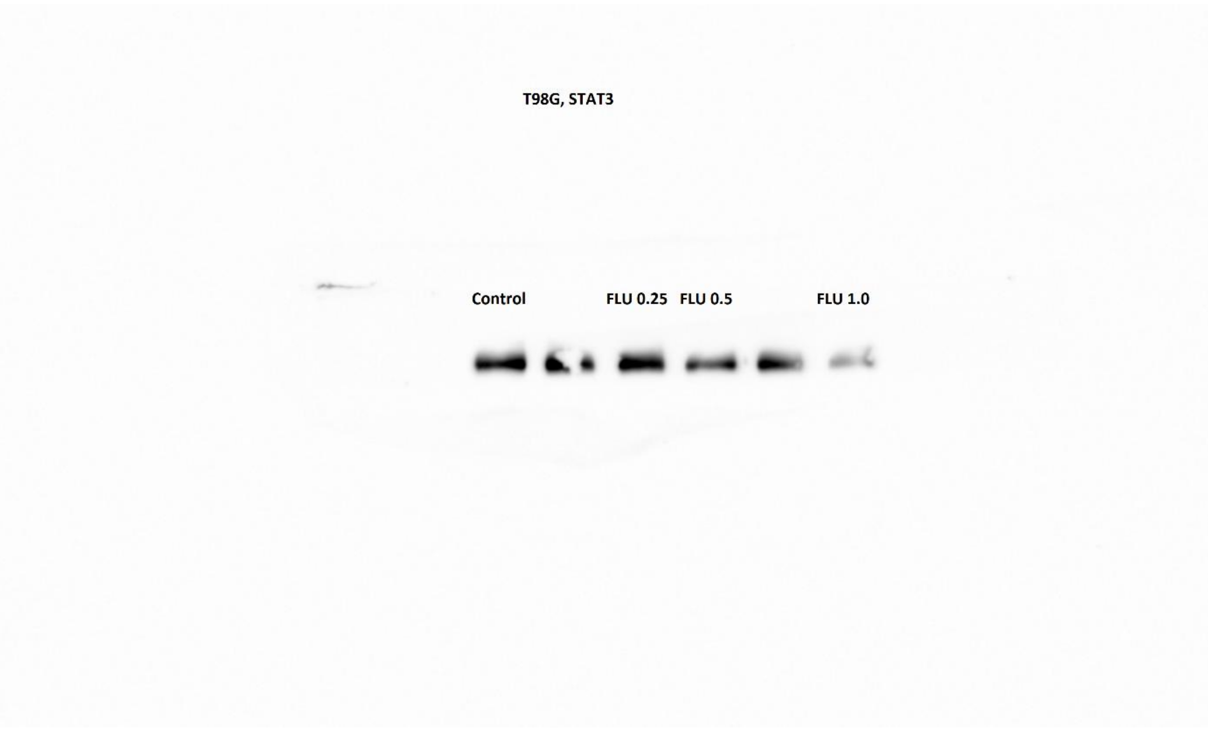
T98G, STAT3
Control
FLU 0.25
FLU 0.5
FLU 1.0

**Original western blot files for figure 3C**

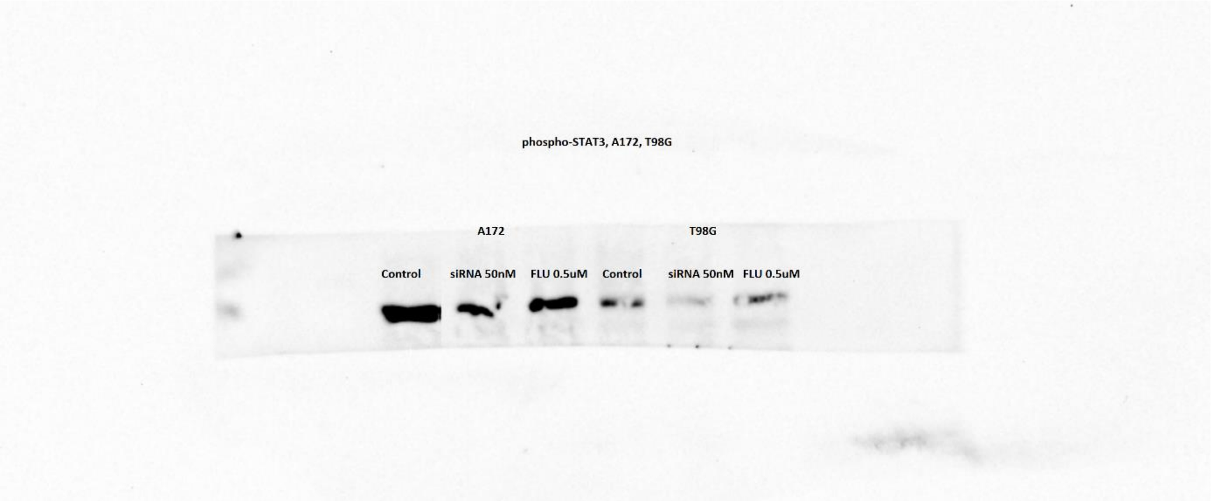


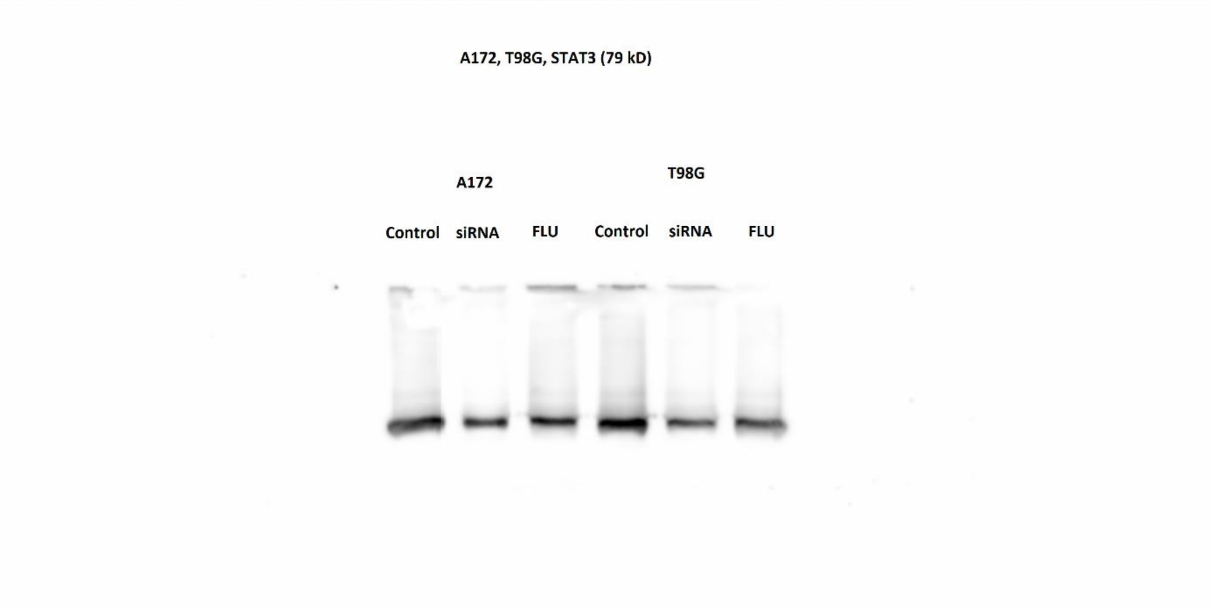


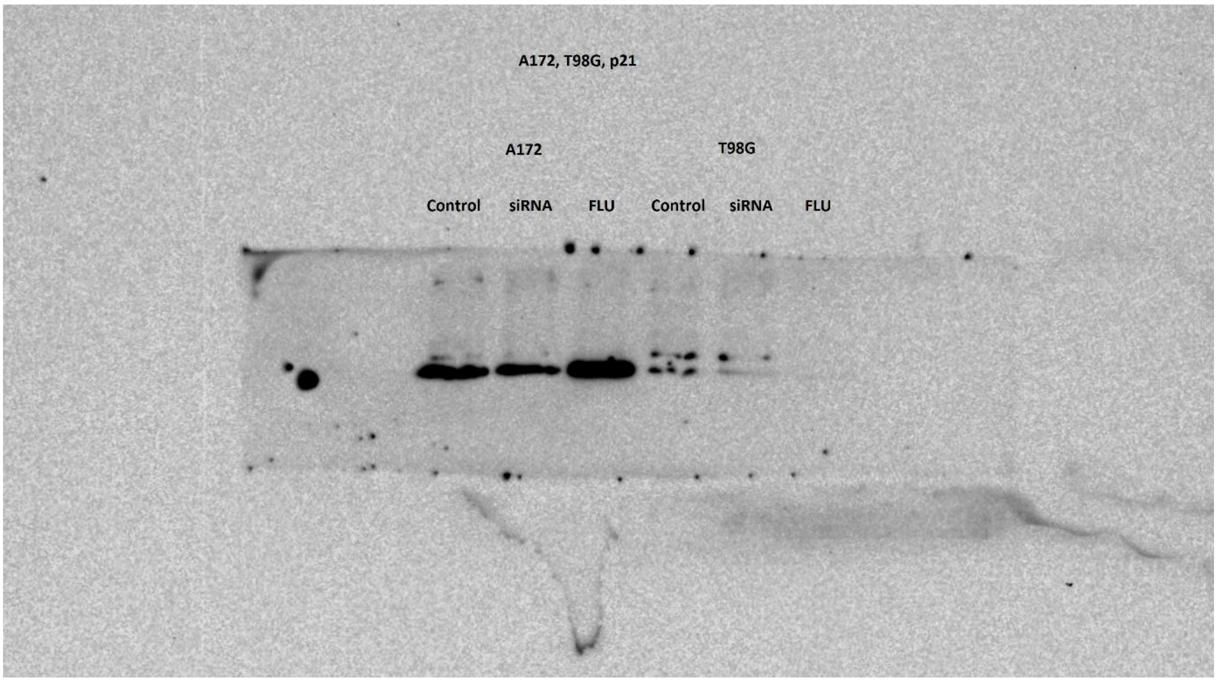

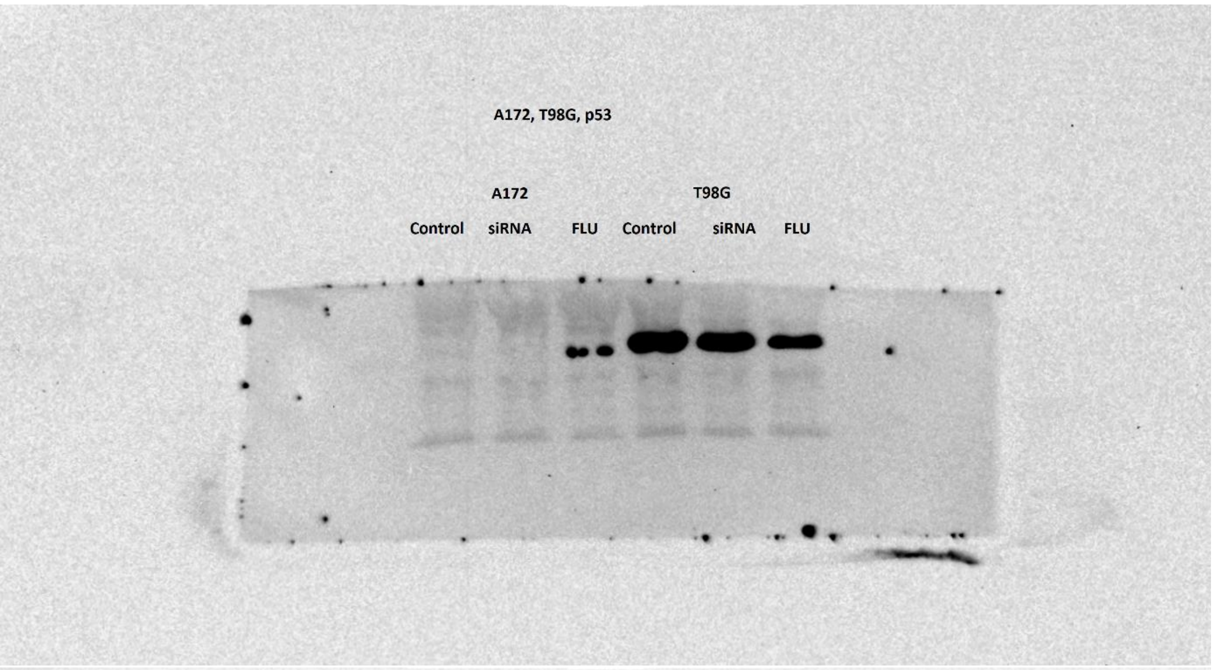
A172, T98G, p53
A172
T98G
Control
siRNA
FLU
Control
siRNA
FLU

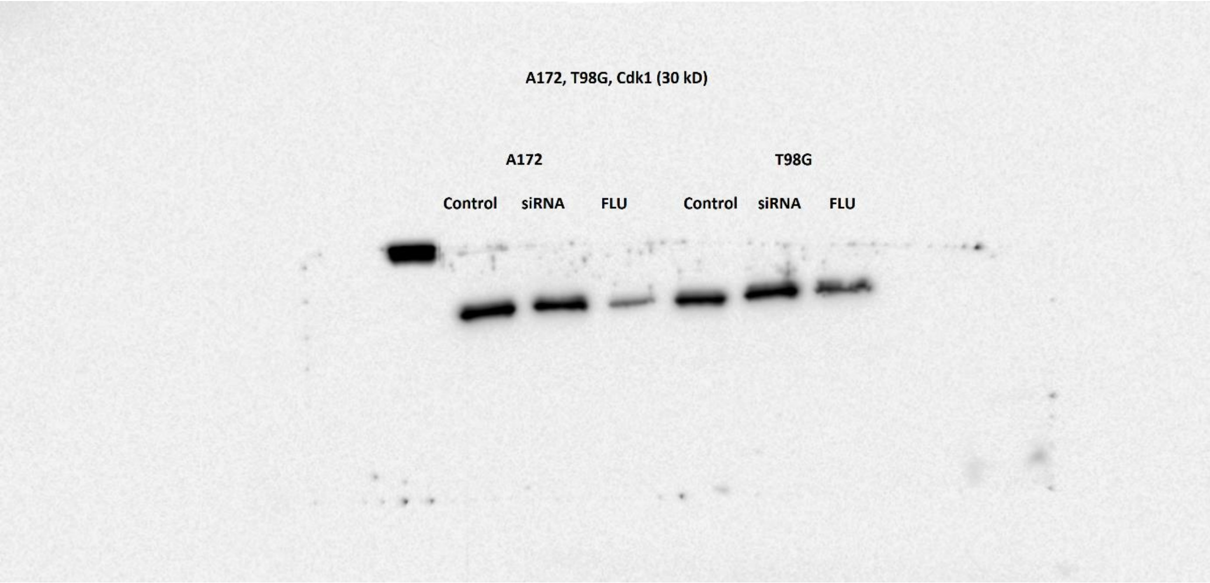
A172, T98G, Cdk1 (30 kD)
A172
T98G
Control
siRNA
FLU
Control
siRNA
FLU

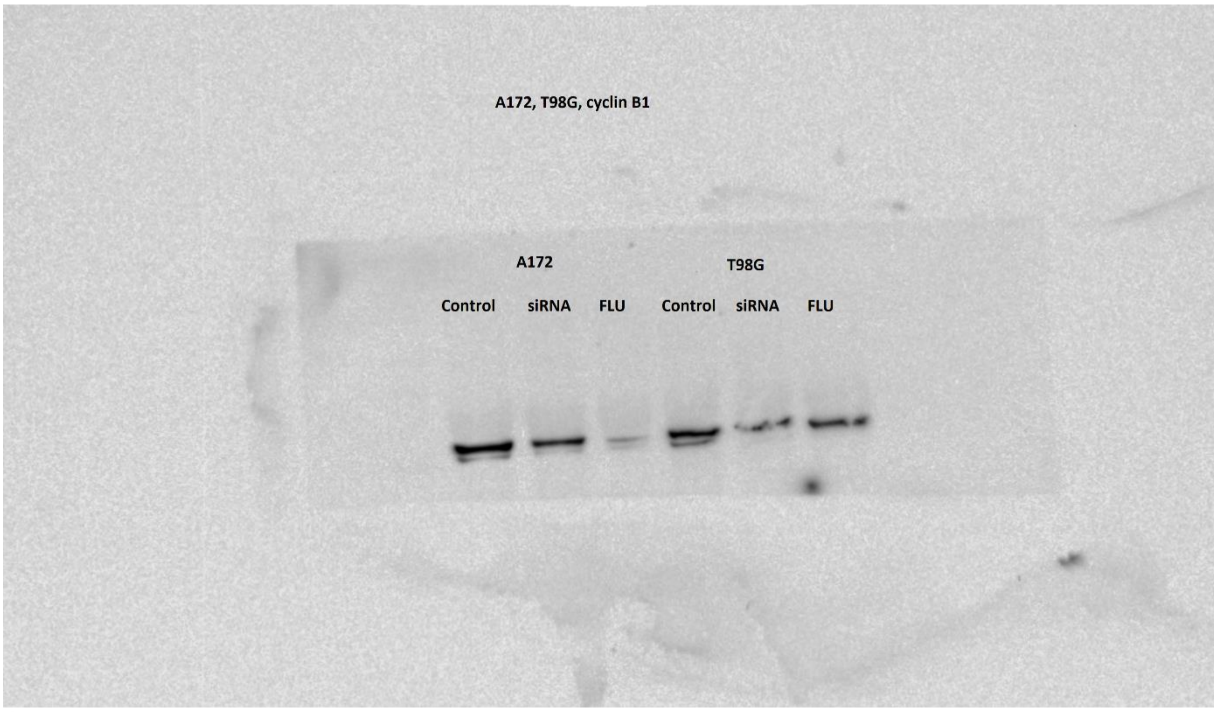
A172, T98G, cyclin B1
A172
T98G
Control
siRNA
FLU
Control
siRNA
FLU

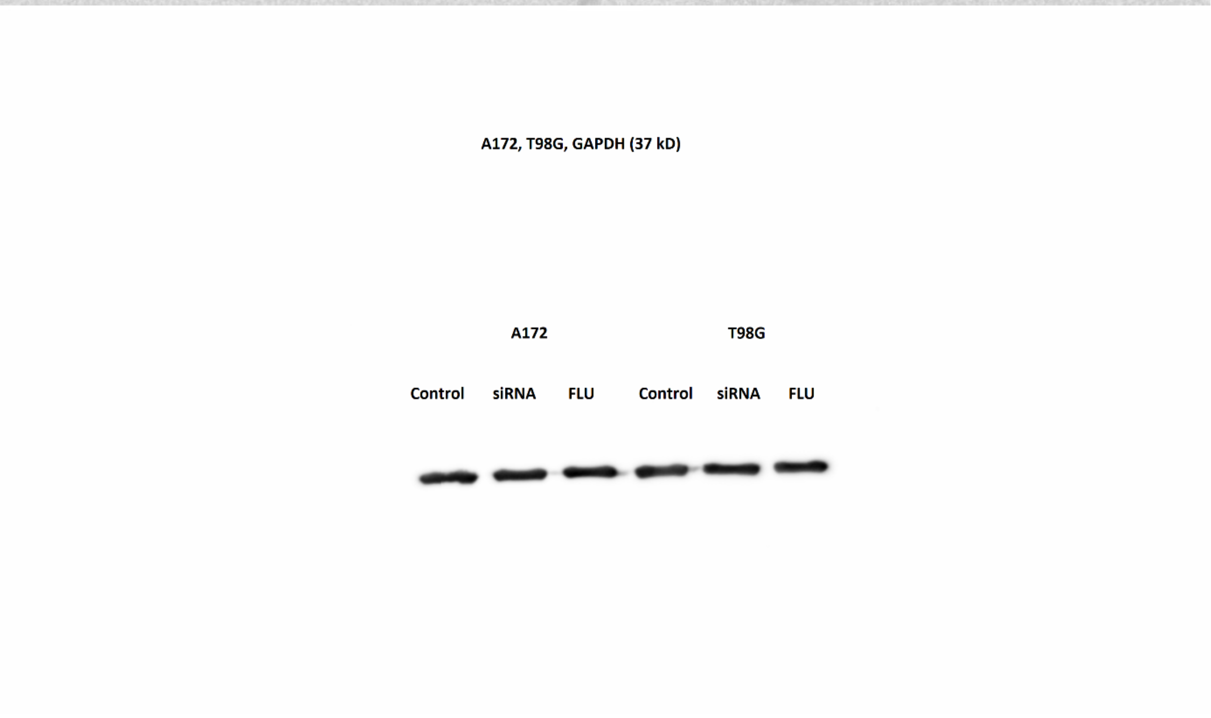
A172, T98G, GAPDH (37 kD)
A172
T98G
Control
siRNA
FLU
Control
siRNA
FLU

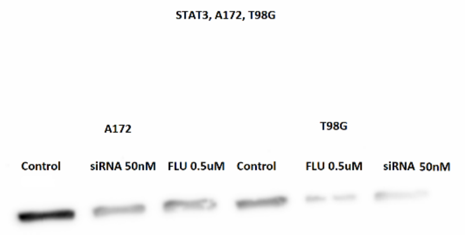
STAT3, A172, T98G
A172
T98G
Control
siRNA 50nM
FLU 0.5uM
Control
FLU 0.5uM
siRNA 50nM

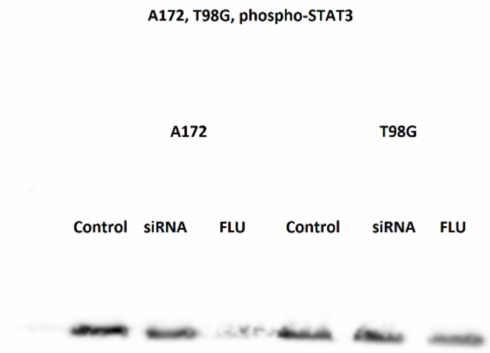
A172, T98G, phospho-STAT3
A172
T98G
Control
siRNA
FLU
Control
siRNA
FLU

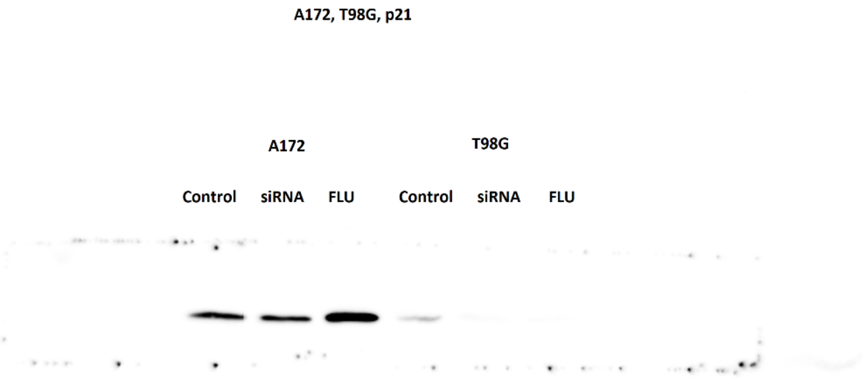
A172, T98G, p21
A172
T98G
Control
siRNA
FLU
Control
siRNA
FLU

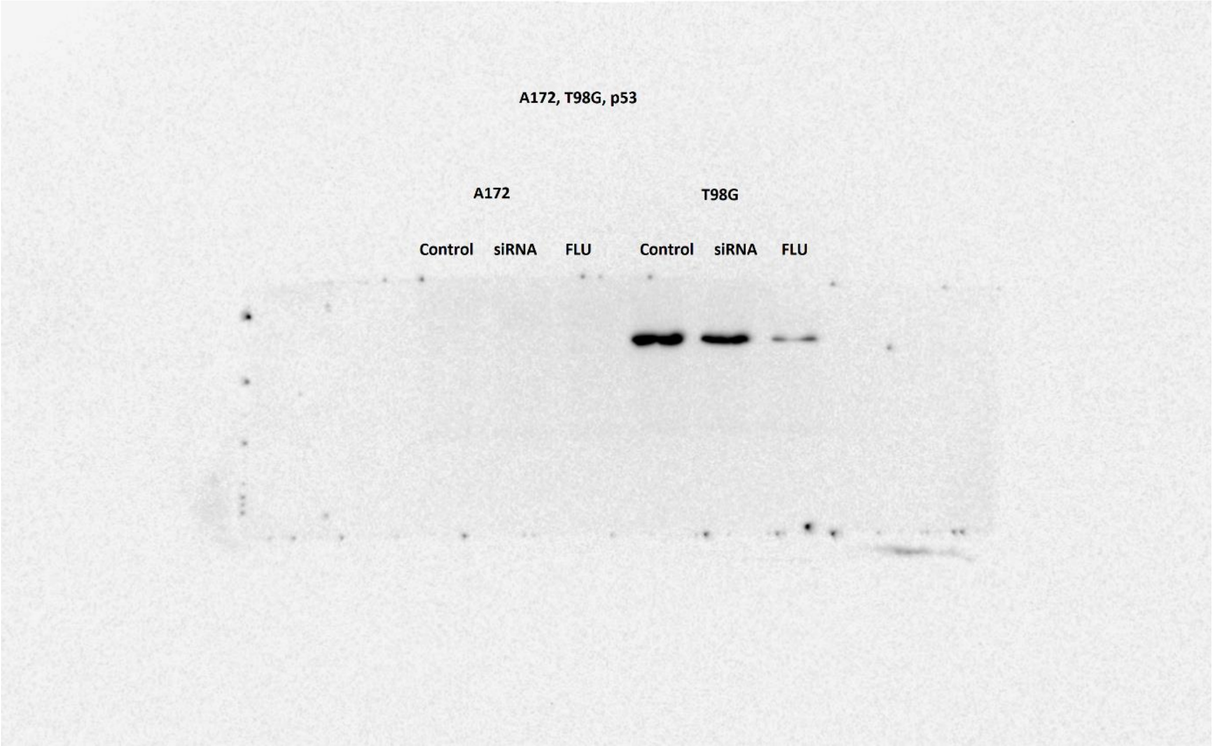
A172, T98G, p53
A172
T98G
Control
siRNA
FLU
Control
siRNA
FLU

A172, T98G, cyclin B1

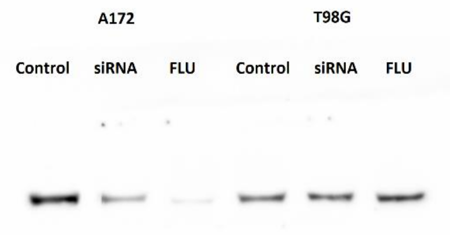


Cdk1, A172, T98G

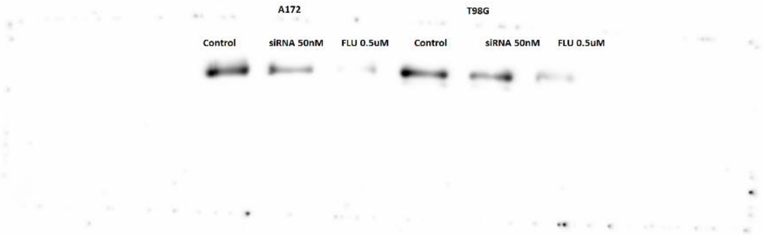

GAPDH, A172, T98G

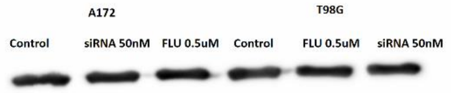


GAPDH, A172, T98G

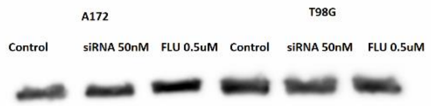

## Original files for figures 2C and 5

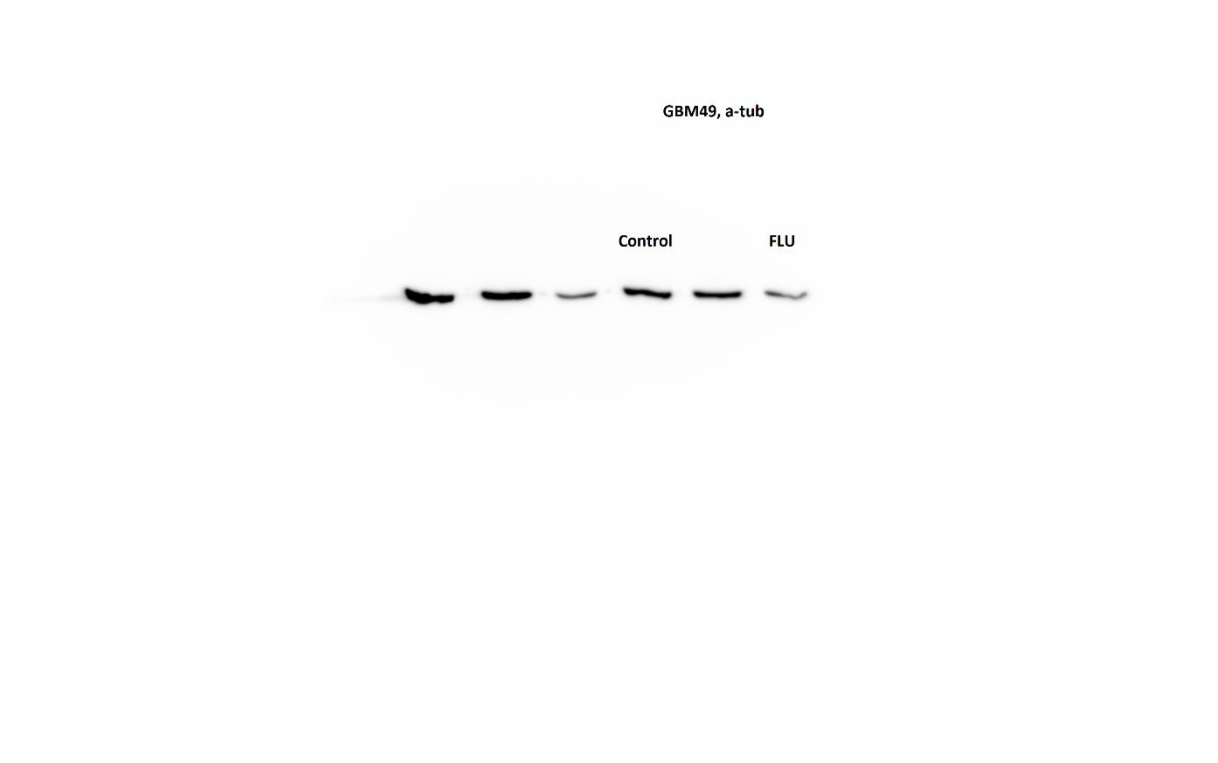


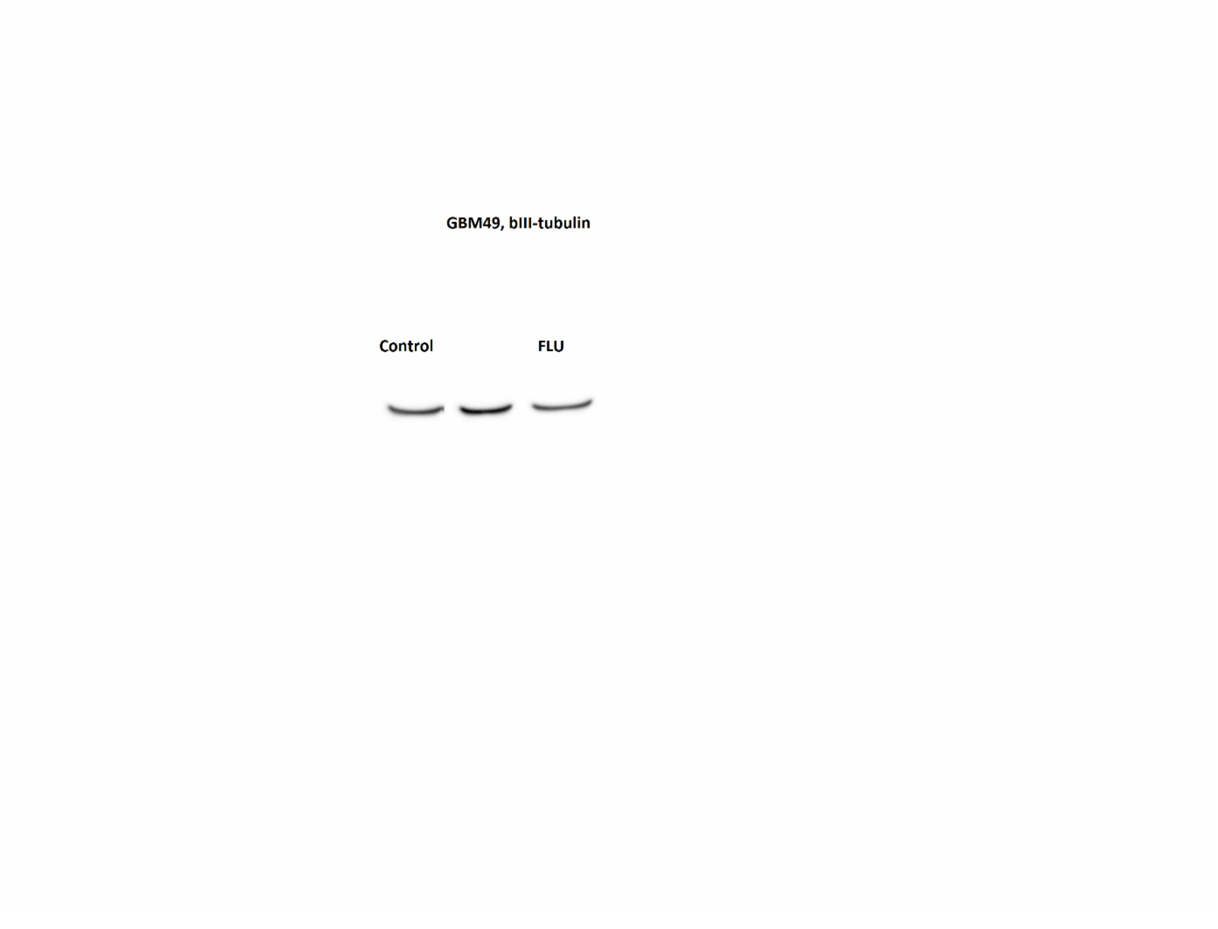

GBM49, GAPDH

Control FLU

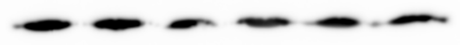

GBM49, STAT3

Control FLU

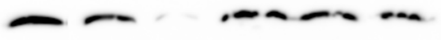

GBM50, a-tubulin

Control FLU

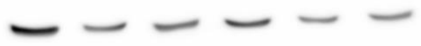

GBM50, bIII-tubulin

Control FLU

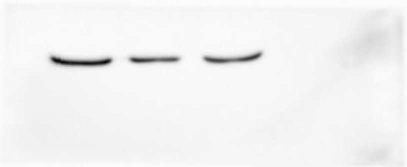

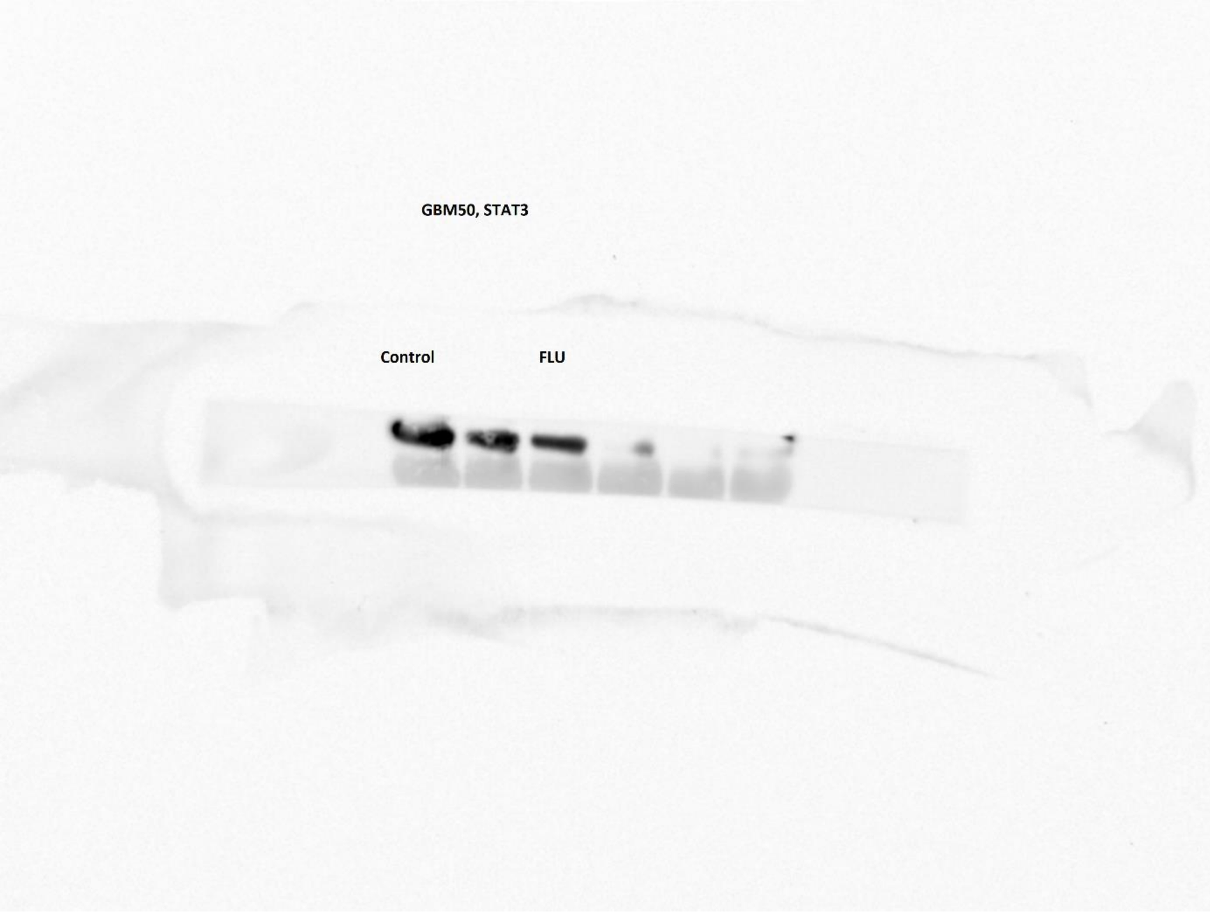
GBM50, STAT3
Control
FLU

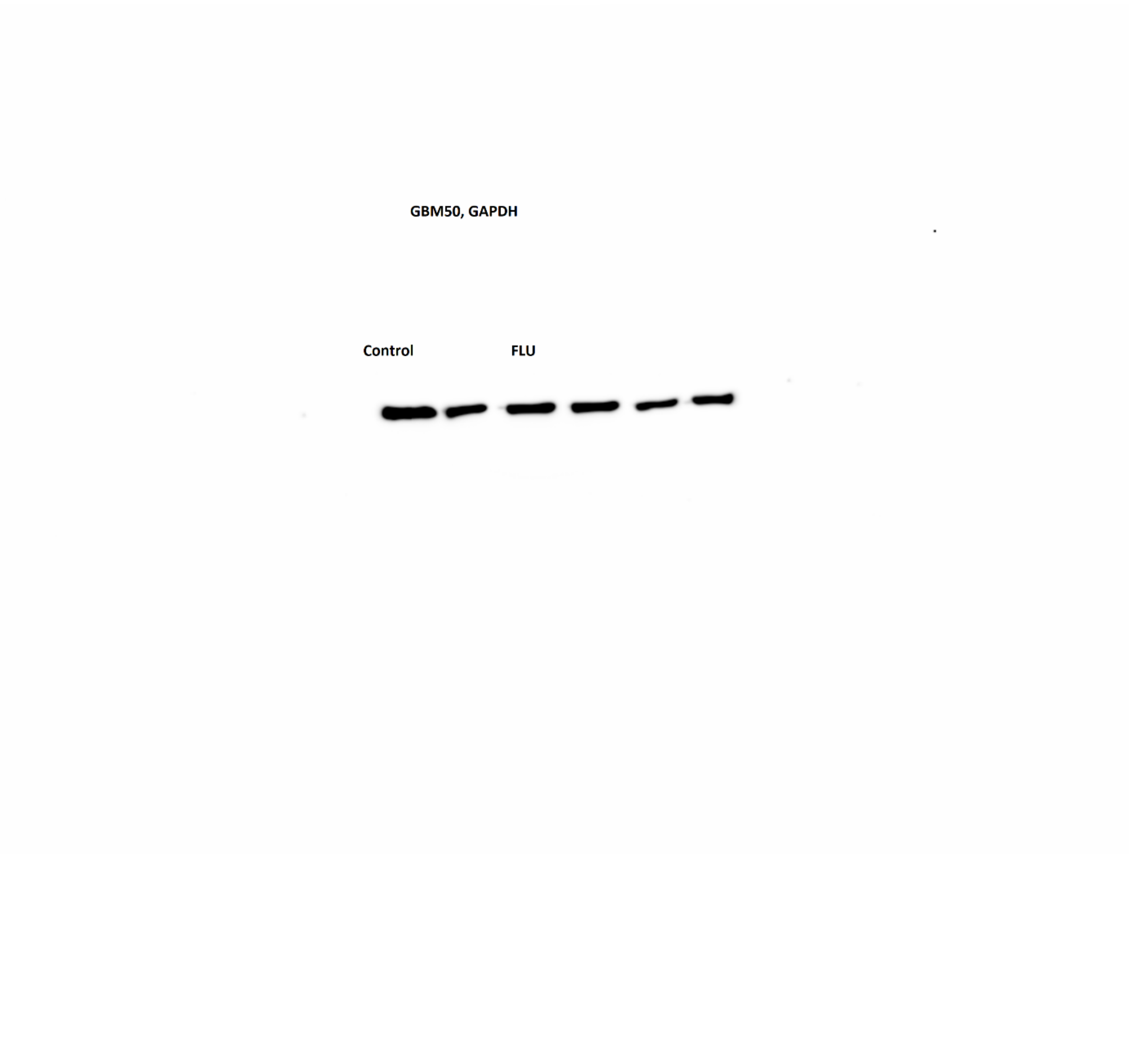
GBM50, GAPDH
Control
FLU

GBM57, a-tubulin

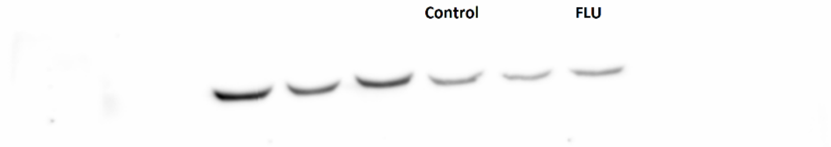


GBM57, bIII-tubulin

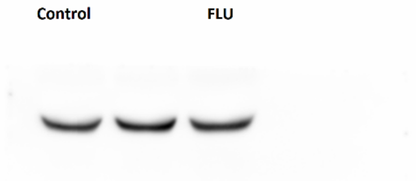

GBM57, STAT3

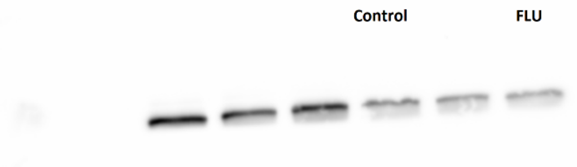


GBM57, GAPDH

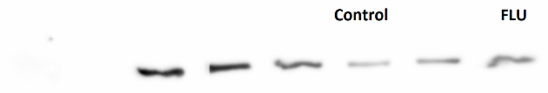

GBM49, GBM50, GBM57, a-tubulin

GBM49 GBM50 GBM57

Control FLU Control FLU Control FLU

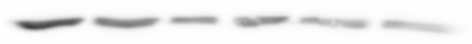

GBM49, GBM50, GBM57, bIII-tubulin

GBM49 GBM50 GBM57

Control FLU Control FLU Control FLU

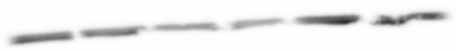

GBM49, GBM50, GBM57, STAT3

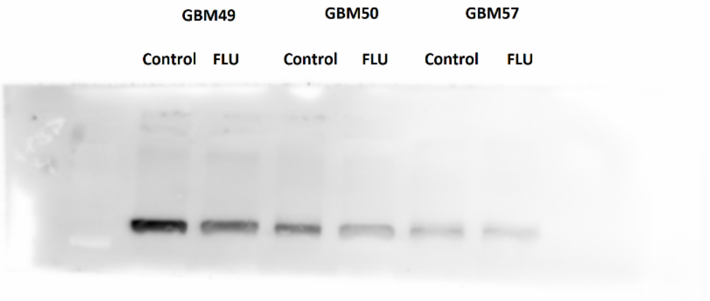


GBM49, GBM50, GBM57, GAPDH

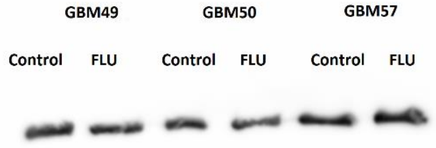

GBM71, GBM72, a-tubulin

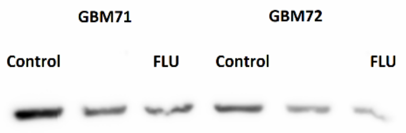


GBM71, GBM72, bIII-tubulin

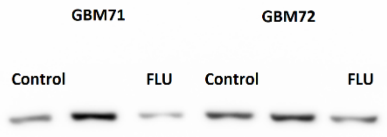

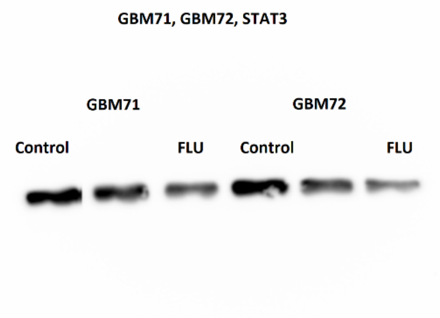
GBM71, GBM72, STAT3
GBM71
GBM72
Control
FLU
Control
FLU

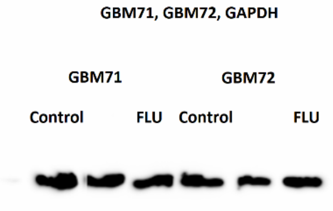
GBM71, GBM72, GAPDH
GBM71
GBM72
Control
FLU
Control
FLU

**GBM73, a-tubulin**

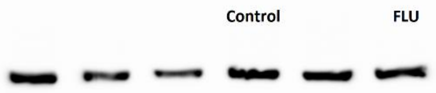


**GBM73, bIII-tubulin**

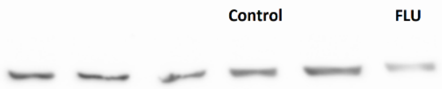

GBM73, STAT3

Control FLU

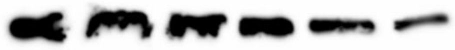

GBM73, GAPDH

Control FLU

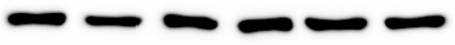

GBM71, GBM72, GBM73, a-tubulin

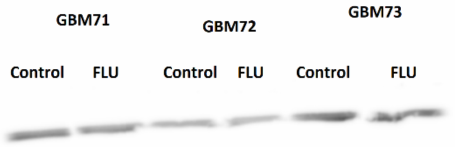


GBM71, GBM72, GBM73, bIII-tubulin

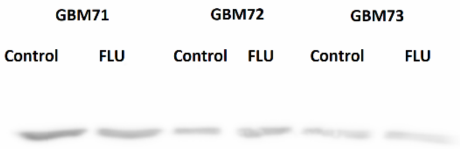

GBM71, GBM72, GBM73, STAT3

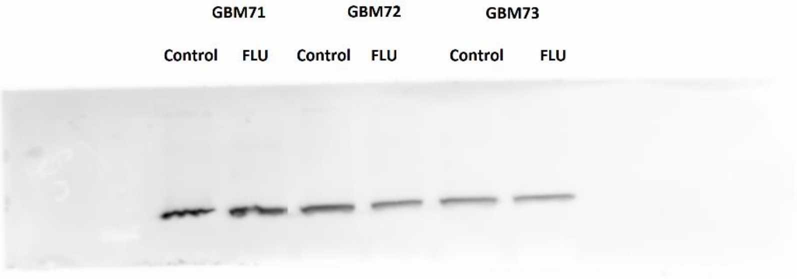


GBM71, GBM72, GBM73, GAPDH

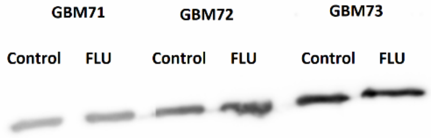

Supplement: Supplementary file 2 — Supplementary Information 2. [file 41598_2023_33047_MOESM2_ESM.pdf]
